# The role of public wheat breeding in reducing food insecurity in South Africa

**DOI:** 10.1371/journal.pone.0209598

**Published:** 2018-12-31

**Authors:** Lawton Nalley, Bruce Dixon, Petronella Chaminuka, Zwiafhela Naledzani, Matthew James Coale

**Affiliations:** 1 Department of Agricultural Economics, Division of Agriculture, University of Arkansas, Fayetteville, Arkansas, United States of America; 2 South African Agricultural Research Council, Pretoria, South Africa; Kansas State University, UNITED STATES

## Abstract

Although classified as an upper middle-income country, food insecurity is still a concern throughout South Africa, as was evident in 2014–2015 when a drought left 22% of households food insecure. Further, a range of domestic and international factors make the local currency unstable, leaving South Africa exposed to risk in global wheat and exchange rate markets and increasing its food insecurity vulnerability. As such, agricultural research in South Africa is needed specifically in plant breeding to increase yields and help mitigate future food insecurity. To foster scientific innovation for food security, the South African government funds the Agricultural Research Council (ARC), which conducts holistic research on wheat and other crops. This study estimates the proportions of increases in yield of ARC’s wheat cultivars, which are attributable solely to genetic improvements. In total, 25,690 yield observations from 125 countrywide test plots from 1998 to 2014 were utilized to estimate the proportions of yield increases attributable to the ARC. We found that South African farmers who adopted the ARC’s wheat varieties experienced an annual yield gain of 0.75%, 0.30%, and 0.093% in winter, facultative, and irrigated spring wheat types, respectively. Using observed area sown to ARC varieties, we estimated that wheat producers gained $106.45 million (2016 USD) during 1992–2015 via the adoption of ARC varieties. We estimated that every dollar invested in the ARC wheat breeding program generated a return of $5.10. Assuming the South African per capita wheat consumption is 60.9 kg/year, our results suggest that the ARC breeding program provided an average of 253,318 additional wheat rations from 1992–2015. Further, the net surplus (consumer plus producer) from the ARC breeding program was estimated at 42.64 million 2016 USD from 1992–2015. Public breeding programs, especially those focused on wheat and other staple foods, must continue if South Africa is to meet growing global food demand, decrease present global food insecurity, and maintain the genetic enhancements that directly enhances yield and benefits low-income consumers.

## Introduction

Since the end of Apartheid in 1991, South Africa has made substantial progress in reducing domestic food insecurity. In October 1994, five months after he was democratically elected to lead South Africa, Nelson Mandela said, “[South Africa’s] principal goal is a better life for all South Africans: black and white, farmer and farm-worker.. .. I would like to give [South Africans] the assurance that the government regards a healthy agricultural sector as indispensable for the continued welfare of South Africa” [1]. For 1994, over 40% of South Africans surveyed with children in their household indicated their children were always or often hungry, compared to just 11% in 2007 [2]. Apart from the increased spending on social welfare programs for the poor, food insecurity has also been reduced by investment in agricultural research and development.

Despite the significant improvements to food security in the early 21^st^ century, food insecurity spiked in November 2015 due to South Africa’s worst drought in 23 years. It was estimated that between November 2014 and November 2015, 22% of South African households had no money to buy food [3]. This proportion reached as high as 41% in the Northwest province and 32%, 31%, and 26% in the Eastern Cape, Northern Cape, and the Free State, respectively. The increased proportions of the hungry were driven by cereal prices (mainly maize and wheat) rising by an estimated 53.7% for the same time period [3]. Elsewhere in the Southern Africa region, food insecurity spiked because of the 2015 drought, leading to an estimated 41.4 million people becoming food insecure [4]. Continued agricultural research is needed, particularly in plant breeding, to increase yields per hectare, breed for biotic and abiotic stresses, reduce yield variability, and help mitigate future food insecurity. The need for increased agricultural research is not unique to South Africa since there is widespread consensus that agricultural research and development are pivotal to economic progress in sub-Saharan Africa’s overall economic growth [5,6, 7].

In South Africa, wheat is the second most consumed grain crop behind maize and is a staple food for the majority of the population living in semi-rural and urban areas [8]. Since 1990, South Africa has consistently imported wheat, mainly due to a decrease in total production area [9]. Given South Africa’s dependence on imports and the recent, significant depreciation of the rand against the USD (by 58% in the last five years [10]), increasing yields per hectare could play a major role in breaking the dependency on imported wheat and helping to alleviate food insecurity by lowering domestic prices. Rising food prices, specifically maize and wheat, which are the staple crops of South Africa, pose a serious problem for both urban and rural poor attempting to combat food insecurity in South Africa [2]. Increases in wheat yield efficiencies in South Africa could also help regional food security, since South Africa is the second largest wheat producer in Sub Saharan Africa behind Ethiopia. In 2014, South Africa was the largest wheat exporter to Zimbabwe, Botswana and Namibia, indicating that yield gains experienced in South Africa can also have spillover effects to regional food security [11].

Increases in wheat yields per hectare could play a large part in eliminating food insecurity, since per capita wheat consumption in South Africa has been estimated to have increased by 1.8% and 8.9% between 1994 and 2009 and 1999 and 2012, respectively [12]. However, previous studies [13, 14, 15] have shown a deceleration in world wheat yield growth (per hectare) since the 1980s, specifically in irrigated areas, which cover approximately 21% of the total wheat production area in South Africa [16]. Although some studies [17,18] have detected a genetic yield plateau in wheat, other studies [19, 20, 21, 22, 23] have found increasing global wheat yields in a linear fashion.

The Food and Agricultural Organization (FAO) estimates that to feed the growing global population, total wheat output (via increased areas planted or genetic gains) would need to increase by 38% (0.86% annually) or 24 kg/ha/year to meet estimated demand in 2050. To put these needed gains in perspective, [24] conducted a meta-analysis of the genetic contributions of global wheat breeding programs. They analyzed twelve wheat-growing environments distributed across the world and found that from 1970 to 2010 global wheat yield potential was rising at only 0.61% annually, less than the 0.86% growth necessary to match demand increases estimated by FAO. Furthermore, they found the gains in spring wheat (the predominate wheat in South Africa) grew at 0.58% annually and winter wheat at 0.70%. They also concluded that there were no differences in average proportionate gains between dry and irrigated wheat. All the studies analyzed by [24] were found to contain strong linear growth patterns, indicating a constantly increasing (but at a decreasing percentage rate) growth in yields. Furthermore, they state that “in order to secure future food supplies, it is essential that the current low rates of progress in yield potential of wheat be accelerated.” They found that of all the major food crops (wheat, rice, maize, soya, and cassava), wheat has shown the lowest rate of progress in yield potential despite its growth in demand as a food crop.

The quality standards for the release of new wheat cultivars in South Africa have been strict since wheat market deregulation in 1997. South Africa has historically produced high-quality wheat due to its high standards for varietal release [25]. South African millers then blend the high-quality South African wheat with lower quality imported wheat to obtain the blend suitable for bakeries. Quality and wheat yields are highly correlated, although negatively, which makes wheat quality an essential consideration in breeding [26]. Wheat yields in South Africa could have increased by 12.81% –19.03% if the focus of South African wheat breeders shifted toward yield gains instead of ensuring newer varieties that met the strict quality standards imposed on new varieties [25].

This study determines what parts (proportions) of observed yield increases in released spring, facultative, and winter wheat cultivars are attributable to genetic improvements by the South African Agricultural Research Council’s Small Grain Institute (ARC/SGI). A total of 36,507 yield observations from 125 test plots across South Africa from 1998 to 2014 are used to model yield gains. The dataset includes 26 ARC/SGI-released varieties (16 spring, 5 facultative, and 5 winter) commercially released to the public between 1992 and 2012. This study also determines if newer cultivars are associated with higher yield variation. Critics of modern varieties (MVs) have suggested that MV yields, although higher, vary more from season-to-season than traditional wheat varieties, thereby exposing consumers and producers to greater production/price volatility. Lastly, we calculate the economic benefits to South African wheat producers from adopting ARC wheat cultivars and the additional rations of wheat made available to South African consumers from the increased yield.

Like many high-income countries the area sown to publically bred wheat varieties in South Africa is diminishing as the number of privately bred, often more expensive, varieties released increases. Unlike most high-income countries, South Africa has many small and impoverished producers who can access publically released varieties. Previous research [27] has shown that given the reluctance of the private breeding sector to address the needs of marginal farmers, the public sector needs to play an active role in research and development, seed production and technology dissemination, especially with adequate support from appropriate government policies. That being said, public breeders such as the ARC must demonstrate tangible benefits to producers and consumers to justify scarce public funding.

This study is the first of its kind in South Africa as it uses a robust (36,507 observations) countrywide panel data set to estimate the gains from wheat breeding. This study is relevant because agricultural research and development projects must compete for funding with other projects that could increase the quality of life in South Africa. Further, the ARC 2015/2016 annual report shows that the total (real) investments in ARC wheat breeding have been declining since 2004. Increased information on the economic impact of wheat cultivar improvements would allow government and private donors to better gauge returns to investments in terms of benefits to consumers (increased wheat rations) and producers (increased revenue). To ensure future funding, the ARC, and other publicly funded research organizations in Africa whose cultivars are released to help low-income producers and consumers, need to provide tangible economic benefits attributable to their modern variety lines. As southern Africa continues to struggle with food insecurity, studies like this can give policymakers and scientists insight on the progress made and distance needed to go to eliminate food insecurity.

### Literature review

The Agricultural Research Council’s Small Grain Institute was founded in 1970 and conducts holistic research on wheat, oats, barley, and triticale. In keeping with its founding objectives, the Small Grain Institute (SGI) has conducted research for the public in areas including plant breeding, soil cultivation, pest and disease control, quality improvement work, and farmer training since the 1970s. Like other global breeding programs, the ARC breeding program focuses on three major breeding components: yield enhancement, quality improvement, and pest and disease resistance (maintenance breeding). Since its creation, the ARC has commercially released 43 wheat varieties at a rate of 1.2 cultivars per year [28] Moreover, the SGI has continually conducted maintenance breeding for evolving diseases and fungi that plague South African wheat production, like stripe rust and crown rot, and pests like the Russian wheat aphid (*Diuraphis noxia*).

Funding for the ARC comes primarily from three sources: The South Africa Parliamentary Grants, external income (revenue derived from project contracts, research and development contracts, and the sale of farm products), and other income such as interest received from short-term investments. In 2014, ARC funding came from the three sources above in proportions of 68%, 30%, and 2%, respectively. Like all public, agricultural research centers, the ARC continuously lobbies for funding. According to the ARC annual report [29], real total revenue declined by 8% due to reduced allocations of Parliamentary Grant from the government from 2014 to 2016 and a lack of growth in private sector investments in agricultural research and development over the last decade. This reduction in research funding is not unique to South Africa. After adjusting for the rising costs of research and inflation, 39% of Sub-Saharan African countries spent less on public food agricultural research and development in 2011 than in 1980 [5].

With evidence [6, 30, 31] available to support the claim that investment in agricultural research and development pays for itself, it is counterintuitive that public funding for agricultural research is decreasing, particularly in the low- and middle-income world. In their comprehensive study [32] found that total public real expenditure on agricultural R&D to total agricultural GDP in 44 low-income countries globally declined from 1980 to 2002 [32]. The results showed that in 2000, the share of agricultural R&D expenditures in agricultural GDP in Africa and Asia was between 0.5% and 0.9%, and Latin America’s share was at 0.98%. These rates are low compared to 2%–3% in high-income countries. Further, [32] show that, in real terms, public expenditure for agriculture increased over the period analyzed; however, agricultural expenditures as a proportion of total government spending showed a declining trend. Studies, such as this one, which highlight the holistic benefits of agricultural research are necessary to provide tangible metrics to policy makers and funding organizations about the benefits of investing in agriculture.

Literature evaluating genetic gains amongst wheat breeders in South Africa is sparse and, with regards to food security gains, is non-existent. In the most comprehensive genetic enhancement cultivar study to date [33], found that the estimated genetic gain in yield potential for spring wheat from 1995 to 2010 grew at 1% annually in the dryland areas of the Western Cape, Ruens and Swartland, South Africa. However, [33] found there was no yield increase observed in cultivar trials. Conversely, [33] found that between 1995 and 2010, winter wheat yields in the Free State of South Africa increased by 0.55% annually due to genetic enhancements. The author also found spring irrigated yields grew at 0.7% annually—solely attributed to genetic gains—over the same time period. The study concludes that spring dryland wheat experienced no genetic gains yield gains from 1995 to 2010, while spring irrigated and winter wheat experienced 0.7% and 0.55% annual yield gains, respectively.

Using a technological k-shift parameter with indexes of varietal improvements [28] estimated the benefits associated with research conducted by the ARC in the South African wheat sector. The k-shift methodology calculates the growth in yield levels due to varietal improvement attributable to research after accounting for other factors contributing to output growth over time. While [28], is the seminal research effort on South African wheat breeding, the authors state that one of the largest drawbacks to their methodological approach was the fact they did not look at empirical test plot yields, only countrywide macro level yields. The authors [28] do specify that “using a regression model to estimate yield indexes, given the required information is available, is definitely a possibility for future studies.” As such, this study builds on the study by [28] by utilizing a large, robust dataset (25,690 individual test plot yields) to estimate the genetic benefits attributed to the ARC wheat breeding program in terms of both yield and yield variance (risk) between 1992 and 2015.

## Materials and methods

**Fig 1:**
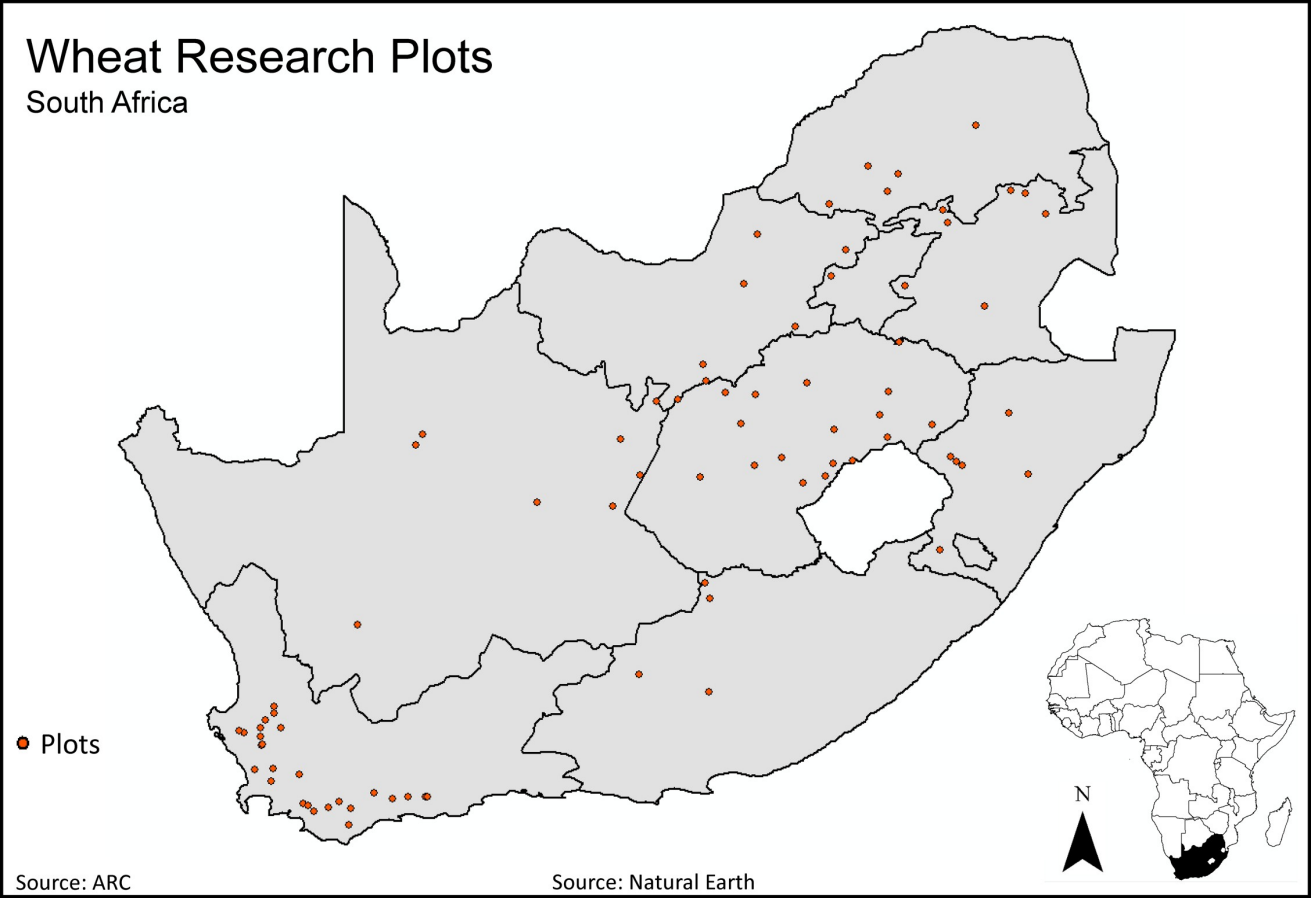
Location of Agricultural Research Council (ARC) wheat cultivar trial locations used in the dataset: 1998–2014.

Wheat is grown in three South African production regions: winter-/spring-planted wheat in the summer rainfall region (Free State province), winter-planted wheat types under dryland conditions within the Mediterranean climate of the Western Cape Province, and spring wheat types grown under irrigation in the Free State’s summer rainfall region [34]. Wheat test plot data were collected from ARC test plots (1.5 x 5 m) throughout 125 plots across South Africa from 1998 to 2014 (Fig 1). Permission from the South African ARC was given to use the dataset in its entirety. A total of 25,690 yield observations were deemed usable from 125 test plots, which included wheat grown under both irrigated and dryland conditions. The dataset included 26 ARC/SGI-released varieties (Table 1), of which 42% were spring wheat (21,643 observations), 41% facultative wheat (10,577 observations), and 17% winter wheat (4,287 observations) varieties grown under both irrigated and dryland conditions (55% and 45%, respectively). Facultative wheats can be sown in winter or spring wheat conditions and generally have less cold tolerance, undergo a shorter but distinct period required for vernalization, and start growing and initiate flowering earlier compared to true winter wheats. The planting dates for spring, winter, and facultative wheat varieties are the same because the diverse geography of South Africa allows for both winter and spring wheat production simultaneously in various parts of the country. Average yields for each wheat type, year, release year by varietal type, and location are reported in S7, S8, S9 and S10 Tables and S3, S4, S5 and S6 Figs.

**Table 1:** Descriptive statistics of South Africa’s Agricultural Research Council’s wheat varieties commercially released between 1992 and 2012.

Variety	Average Yield (kg/ha)	Yield Ratio^a^	Yield Difference (kg/ha)^a^	Coefficient of Variation (%)	Year Released to Public	Type	Observations	SD of Yield	Percentage Irrigated
TUGELA DN	3,123	-	-	55.97	1992	Winter	416	1,748.23	0
BETTADN	2,632	0.84	-491	49.38	1993	Winter	2,070	1,299.82	0.39
GARIEP	2,818	0.9	-306	47.78	1994	Facultative	2,420	1,346.40	0.33
LIMPOPO	2,679	0.86	-445	50.66	1994	Facultative	2,055	1,357	0.39
KARIEGA	5,598	1.79	2,474	40.64	1994	Spring	3,733	2,274.98	66.33
MARICO	6,338	2.03	3,215	33.29	1994	Spring	1,510	2,110.16	99.27
CALEDON	2,780	0.89	-344	46.7	1996	Facultative	2,176	1,298.22	0.37
ELANDS	2,873	0.92	-250	47.6	1999	Facultative	2,407	1,367.65	0.33
STEENBRAS	6,102	1.95	2,979	32.07	2000	Spring	2,273	1,956.85	96.48
BAVIAANS	5,527	1.77	2,403	39.85	2001	Spring	3,305	2,202.30	67.08
KOMATI	2,762	0.88	-361	49.67	2003	Facultative	1,519	1,371.96	0.53
BIEDOU	4,671	1.5	1,548	41.67	2003	Spring	763	1,946.57	48.23
OLIFANTS	6,565	2.1	3,442	30.47	2003	Spring	1,948	2,000.29	98.56
TARKA	2,188	0.7	-935	49.44	2003	Winter	262	1,081.85	3.05
DUZI	6,772	2.17	3,648	30.61	2006	Spring	1,893	2,072.59	94.51
KROKODIL	7,059	2.26	3,935	30.95	2006	Spring	1,793	2,184.77	100
MATLABAS	3,061	0.98	-62	49.6	2006	Winter	1,258	1,518.48	0
NOSSOB	1,732	0.55	-1,391	55.28	2006	Winter	281	957.7	0
BUFFELS	7,276	2.33	4,153	29.62	2009	Spring	833	2,155.46	100
TANKWA	3,682	1.18	558	31.79	2009	Spring	841	1,170.38	0
SABIE	7,475	2.39	4,351	26.96	2010	Spring	843	2,015.39	98.1
KOONAP	2,604	0.83	-520	47.67	2012	Spring	358	1,241.29	0
KWARTEL	3,715	1.19	591	26.65	2012	Spring	434	989.87	0
RATEL	4,016	1.29	892	26.94	2012	Spring	431	1,081.63	0
SENQU	2,891	0.93	-233	48.39	2012	Spring	235	1,398.66	0
UMLAZI	8,021	2.57	4,897	22.9	2012	Spring	450	1,837.05	100

^a^ Yield ratios are relative to Tugela-DN which was the first variety released in the study in 1992.

Under the National Wheat Cultivar Evaluation Program (NWCEP) run by ARC-Small Grains Institute, the wheat test plots were planted in winter each year (May–July) on producers’ fields across South Africa according to a randomized block design with four replicates at each location each year. The trials were planted in one of two periods, normal or late, depending on their location. Late planting is typically for dryland wheat production because this production method typically precedes a fallow period to conserve soil moisture. [35] showed that pre-planting moisture is one of the most important determinants in dryland (rainfed) wheat production. As such, wheat planted after a fallow period may benefit from higher soil moisture, which is highly desirable amongst dryland producers, specifically in the relatively dry Free State. Under irrigated production, there are years when producers harvest maize and then sow wheat (double crop). Climatic conditions dictate when the maize crop is harvested; thus, wheat following maize may be past the optimal planting date. As such, the dataset includes 10,949 late-planting observations to mimic these production conditions/constraints. Average yield observations by normal and late planting dates are shown in S2 Fig.

Seeding rates of each cultivar were calculated according to recommendations by ARC breeders. Fertilizer was applied according to recommendations for the area and, in most cases, by the farmer together with the rest of their wheat crop. Although cultural practices vary somewhat across participant and production locations, each wheat trial was produced under typical farming practices for each given region, and all trials were visited by ARC staff to monitor growth and production practices. S1 Fig shows how average annual yields (kg/ha) observed in ARC field trials correlate with actual yields (kg/ha) reported in South Africa by FAOSTAT for the same time period. S1 Fig highlights the high correlation in relative yields between on-farm and experimental test plot yields across time.

Although a gap between experimental and actual yields exists, [36] concluded that the most reliable sources of relative yields are cultivar trials compared with producer field observations. So, despite yields often being greater in experimental test plots compared with producers’ fields, the relative yield differences between varieties can be compared. We assume the same yield gains observed in varieties on ARC experimental plots are also captured by wheat producers across South Africa.

The Just-Pope production function offers flexibility in describing a stochastic production function that can exhibit changes in the mean and variance of output [37]. This method provides a straightforward procedure for testing if the determinants of increased yield also affect yield stability. Specifically, the Just-Pope production function allows the inputs to affect both the conditional mean and conditional variance of the outputs where the mean and variance functions are conditioned on a set of exogenous factors that can vary by observation. An advantage of the Just-Pope production function is that heteroscedasticity is explicitly incorporated into the model. This is important for varietal traits because trait variations due to breeding (yield, disease resistance, etc.) likely alter variances around variety-specific means, implying heteroscedasticity. Wheat is grown throughout heterogeneous areas across South Africa and varieties are specifically bred for resistance to different pathogens and insects and adaptation to various agronomic conditions. This implies a strong likelihood that variances around different cultivar means could be heteroscedastic.

The main statistical challenge in using field trial data is typically the same varieties are not included in every trial year, due to the entry and exit of varieties across time. Genetic improvement is captured by identifying the mean yield for each variety and then plotting these estimates against each variety’s release year (RLYR). The RLYR of each variety can be interpreted as the “vintage” of a breeding technology [38, 39, 40]. The coefficients on RLYR (one for the conditional mean function and one for the conditional variance function which gives the variance of the regression error term) capture the progression of the wheat breeding technology across time (conditional mean function) and yield variance of the ARC wheat breeding program (conditional variance function). However, a distinction exists between RLYR, which varies from 1992 to 2012, and the field plot trial dates, which vary from 1998 to 2014. Each variety has a single RLYR, the date that the wheat variety was released commercially to the public, and each one embodies the breeding technology that was readily available for that given year. In the estimated regression model, the coefficient on RLYR only captures the effect of wheat seed technology at the specific year of release. A typical life-cycle of a variety begins with a variety producing relatively higher yields than previously released varieties during the early years of adoption and ends with its eventual replacement by yet higher-yielding releases [40]. Average yield by RLYR is shown in S9 Table and S6 Fig.

RLYR is not a time-trend variable but is modeled similarly to the way in which the growth model [38] showed the embodied technology [39]. Specifically, [38] assigned “serial numbers” of ordinal magnitude to the embodied technology in capital. In this model, the variable RLYR represents the embodied technology for a given year of release for a wheat variety by the ARC breeding program. This method is standard procedure for measuring the impact of technological change on output [40]. Separate regressions were estimated for each wheat type (spring, facultative, and winter), since each was bred for different growing environments and, as such, would have different mean yields and yield variances by location and by year.

Importantly, management practices vary by location (irrigated or non-irrigated) and year, as many of the test plots are on actual wheat producers’ fields. Results from a single year or single site could be misleading, due to the possibility of extreme weather events (particularly for dryland wheat production) or pest pressure. Annual and location fixed effects are included in the regression model to account for differences in management and production practices across years and locations. Location fixed effects are included in the model to account for location-specific factors, including time-invariant factors such as altitude and soil texture. Potential yield trends over time are accounted for by including test plot-year fixed effects. Including these fixed effects, the regression models for yield and yield variance, respectively, for a given variety become:

$$Y_{jtw}=\beta_{0}+\beta_{1}LNRLYR_{jtw}+\beta_{2}PLANTING_{jtw}+\delta_{t}+\varphi_{j}+\varepsilon_{jtw},$$ (1)

$$ln{\left(e_{jtw}\right)}^{2}=\alpha_{0+}\alpha_{1}LNRYLR_{jtw}+\alpha_{2}PLANTING_{jtw}+\delta_{t}*+\varphi_{j}{{}^{*}}_{+}\varepsilon_{jtw},$$ (2)

where Y_jtw_ is the yield in kg/ha for plot j in time period t for wheat type w (winter, spring irrigated, spring dry and facultative). PLANTING_jtw_ is qualitative (0–1) indicating if the particular plot was planted late (0) or during its optimal planting period (1). LNRLYR_jtw_ is the log of release year for the yield observed on the plot j in year t and for wheat type w (spring, winter and facultative). Four pairs of (1) and (2) are estimated, one for each varietal type (spring irrigated, spring dryland, facultative and winter) since the breeding goals (drought tolerance, quality, and heat stress tolerance) differ for each.

The term δ_**t**_ represents a vector of coefficients. A given coefficient represents each year (t), from t = 1998 (the year of the first test plot) to t = 2014, with t = 1998 being omitted as the base (default) year. A squared RLYR term was initially modeled to capture curvature in genetic gain, but due to perfect collinearity between RLYR and RLYR^2^, the LNRLYR was used to capture curvature. The term *φ*_*i*_ is a vector of coefficients with each coefficient representing one of the 125 locations, or experiment plots, where the variety test performance experiments were conducted. All dryland and facultative wheat in the study were produced under dryland conditions; however, spring wheat was produced under both dryland and irrigated practices. Thus, separate models were run for spring irrigated and spring dryland wheat as they were bred for different agronomic and climatic environments. A binary variable indicating dryland or irrigated is inserted into (1) and (2) when estimating the equations for spring wheat.

Wheat yields are also a function of climatic characteristics such as precipitation (in the case of dryland wheat), solar radiation, and temperature. Ideally, independent variables representing these forces would enter into the equations estimating yield and yield variance. However, like the majority of large panel datasets from low-/middle-income countries, weather data were not available in their entirety. As such, year (*δ*) and location (*φ*) fixed effects also account for weather impacts. Eqs (1) and (2) are estimated by feasible generalized least squares [40]. Standard errors are clustered by year and the three regions.

## Results

### Winter wheat

The coefficient of RLYR is the focus variable in this study since it captures the “vintage” of each cultivar, i.e., the level of technology that characterizes each wheat cultivar. Using the Just-Pope results from the full fixed effects model (S5 Table) and using β_1_ and LNRLYR for each year to calculate annual average gains, it was found that on average, from 1992 (with the release of Tugela-DN) to 2015, the ARC wheat breeding program added 21.58 kg/ha (P<0.01) annually in their winter wheat varietal releases. Average change is varietal (winter, spring dryland, spring irrigated, and facultative) specific where average gain is calculated as (${\hat{\beta }}_{1}$ $\sum_{t=1}^{T}lnRLYR$) / T, where ${\hat{\beta }}_{1}$ is the respective estimate of β_1_. The model explains 55% of the yield variation, and both Year and plot fixed effects were highly (P<0.01) significant. The variance parameter estimates show that from 1992 to 2014, the varieties released by the ARC Breeding Program experienced some (P<0.05) increase in annual yield variance (S5 Table). This observation would imply that yield risk (as measured by the variance of the Just-Pope model) is increasing over the sample period, indicating that ARC-released winter wheat variety yields have increased with a tradeoff with increased yield variation.

### Spring wheat

Using the Just-Pope results from the model (S3 and S4 Tables) and transforming the β_1_ and LNRLYR into annual average gains, on the average from 1994 (with the release of Marico) to 2014, the ARC wheat breeding program added 9.53 kg/ha (P<0.01) annually with the release of their irrigated spring wheat varieties. The model explains 49% of the yield variation, and both Year and plot fixed effects were (P<0.10) significant. Unlike the findings in the winter wheat varieties, the associated yield gain attributed to the ARC irrigated spring wheat breeding program is not accompanied by an increase in yield variance (P>0.10). This implies producers using irrigated spring wheat released by ARC experienced yield gains with no associated increase in yield risk. The insignificance of the LNRLYR coefficient on spring dryland wheat (S4 Table) could be attributed to the fact that three of the four ARC dryland spring wheat varieties were released in the same year (2012) and the other was released shortly before (2009), resulting in a short window for finding significant progress from breeding technology.

### Facultative wheat

The Just-Pope LNRLYR coefficient estimates associated with facultative wheat (Table 2) indicate the ARC breeding program has added 7.38 kg/ha (P<0.05) annually, on average, since the release of Limpopo in 1994. Again, both the fixed effects for plot and Year were highly statistically significant (P<0.01) with the model explaining 53% of the yield variation. Similar to spring irrigated wheat, the yield gains associated with facultative wheat did not come with greater yield variance.

**Table 2:** Regression results for RLYR coefficients for all South African Agriculture Research Council wheat types.

Wheat Type	OLS Yield	Just-Pope Variance	Just-Pope Yield	Observations	Mean Yield (kg/ha)
Winter	160.99***	0.12**	163.00***	4,287	2,760.2
Spring Irrigated	65.13***	0.02	64.81***	8,527	6,696.4
Spring Dryland	20.82	-0.12	19.79	2,299	3,548.5
Facultative	51.90***	-0.01	52.57***	10,577	2,825

*** (P<0.01)

** (P<0.05)

*(P<0.10)

Full fixed effects regression results for all models run are found on S3, S4, S5 and S6 Tables.

### Cumulative genetic gain

An important feature of the calculation of genetic gains associated with a breeding program is to account for the program’s cumulative effects over the entire period. That is, the yield gains attributable to the breeding program in 1993 are those observed in 1993 plus those observed in 1992. Therefore, the genetic gains for 2015 would be the sum of the year-specific genetic gains from 1992 to 2015 for winter wheat and 1994 to 2015 for spring and facultative wheat, corresponding to the first release of each variety in the study. For example the lnRLYR coefficient on winter wheat is 163.00 (Table 2). The first ARC winter wheat variety was released in 1992 and its gain was equivalent to the natural log of the year (which for estimation purposes was 1, the year of initial release) multiplied by the winter wheat coefficient found on Table 2. Therefore, the cumulative gain for 1993 (or year two) is simply ln(2)*163, or 112.98 kg/ha, as illustrated on column 5 on Table 3. The cumulative gains in any period for a varietal (columns 3, 5 and 7 on Table 3) are actually the sum of the gains (changes) in all periods before and up to the current year. For example, 54.89 in column 2 for 1997 is the sum of (27.44 − 0) + (43.5 − 27.44) + (54.89 − 43.50) = 54.89.

**Table 3:** Cumulative gains of ARC spring, winter and facultative wheat varieties: 1992–2015.

Year	ARC Spring Irrigated Wheat (Ha)^a^	Spring Irrigated Wheat Cumulative Genetic Gain (kg/ha)^b^	ARC Winter Wheat (Ha)^a^	Winter Wheat Cumulative Genetic Gain (kg/ha)^a^	ARC Facultative Wheat (Ha)^a^	Facultative Wheat Cumulative Genetic Gain (kg/ha)^b^	Price USD/Ton	2016 USD Prices/Ton
1992	334,813	-	6,631	0.00	8,066	-	$250.00	$428.88
1993	368,953	-	20,612	112.98	200,382	-	$230.40	$382.12
1994	420,263	0.00	22,218	179.08	148,430	0.00	$212.60	$344.52
1995	400,307	44.92	56,428	225.97	238,609	36.44	$218.60	$344.58
1996	321,059	71.20	35,062	262.34	307,282	57.75	$210.50	$323.00
1997	181,780	89.85	37,713	292.06	229,113	72.88	$177.50	$264.32
1998	27,226	104.03	20,091	317.19	149,071	84.61	$146.20	$215.27
1999	42,564	116.12	9,765	338.95	22,723	94.19	$157.30	$226.61
2000	10,118	126.11	2,148	358.15	6,779	102.29	$167.90	$234.01
2001	29,661	134.77	15,097	375.33	35,954	109.31	$165.20	$223.88
2002	18,065	142.40	15,714	390.86	42,731	115.50	$149.10	$198.92
2003	21,086	149.23	1,122	405.05	27,994	121.04	$188.80	$246.27
2004	30,133	155.41	2,158	418.09	41,746	126.05	$168.90	$214.60
2005	38,185	161.05	6,833	430.17	91,607	130.63	$162.60	$199.82
2006	83,712	166.23	5,776	441.42	33,099	134.84	$225.10	$267.98
2007	62,719	171.04	13,678	451.94	11,808	138.73	$355.70	$411.74
2008	60,867	175.51	11,203	461.82	49,421	142.36	$279.30	$311.35
2009	74,963	179.69	3,010	471.14	15,900	145.75	$189.80	$212.33
2010	44,187	183.62	3,845	479.95	37,396	148.94	$316.10	$347.92
2011	61,520	187.33	2,781	488.31	12,310	151.94	$326.40	$348.26
2012	40,124	190.83	2,149	496.27	5,482	154.78	$354.90	$371.00
2013	23,703	194.15	1,499	503.85	1,652	157.48	$298.30	$307.33
2014	8,231	197.31	1,541	511.09	844	160.05	$281.30	$285.19
2015	930	200.33	2,457	518.03	2,448	162.49	$295.20	$298.92

^a^As derived from S1 Table. South Africa does not reported irrigated versus non-irrigated spring wheat area. As such, it was assumed that the total percentage of spring wheat observations in the dataset which were irrigated (78.77%), was equivalent to the total percentage actually sown by producers in South Africa.

^b^As derived from Just-Pope Yield coefficient on Table 2

Winter wheat had the largest gain by wheat type, 518.03 kg/ha, from the initial release of Tugela-DN in 1992 (Table 3). The average yield of Tugela-DN was 3,123 kg/ha (416 observations), which equates to a 16.58% increase over the initial release of Tugela-DN through 2014 and is solely attributed to the ARC breeding program. This difference would equate to an annual increase of 0.75%. This increase is less than the FAO’s estimate that total wheat output (via increased area planted or genetic gains) would need to increase by 38% (0.86% annually) to meet the demand of feeding the growing global population by 2050.

Cumulative genetic gains through the ARC breeding program for irrigated spring wheat were estimated at 200.33 kg/ha (Table 3) from the initial release of Marico and Kariega in 1994 through the 2014 growing season. The average yield for Marico and Kariega was 6,561.41 kg/ha (4,952 observations) resulting in a 3.05% total increase. The regression coefficient on facultative wheat from Table 2 estimates a cumulative gain of 162.49 kg/ha (Table 3) from the initial release of Gariep and Limpopo in 1994 through the 2014 growing season. The average facultative yield for Gariep and Limpopo was 2,755.51 kg/ha (4,460 observations), resulting in a genetic gain of 5.89% across the entire time period or 0.30% annually.

These results suggest that the largest gains in the ARC are from dryland wheat breeding because all winter wheat observations were under dryland conditions and less than 1% of facultative wheat was irrigated. The average annual gain of dryland (facultative and winter wheats) was 0.72%, which is larger than the percentages in the meta-analysis conducted by [24], which found that global wheat yield potential was only rising at 0.61% annually. Our winter/dryland results are in line with [24], who found that global winter wheat annual yield increases of 0.70%, compared to our 0.72%. However, the gains estimated in this study are still below the 0.86% growth necessary to match demand increases estimated by FAO. Like the study by [24], we find the average annual genetic gains for spring wheat are less than for winter wheat. The results above (dryland and irrigated spring wheat and dryland winter and facultative wheat) are in line with [33] who found that South African spring dryland wheat experienced no genetic yield gains, while spring irrigated and winter wheat experienced a 0.7% and 0.55% annual yield gain, respectively from 1995 to 2010.

With the exception of dryland spring wheat, we find wheat breeding conducted by ARC in South Africa has not reached a genetic plateau. Results from dryland spring wheat need to be viewed with caution, given the relatively few commercially-released varieties in the dataset. Furthermore, S1 Table indicates the majority (99.74%) of spring wheat planted in South Africa is not sown to ARC varieties; in contrast, 19.54% of South Africa is sown to ARC winter wheat varieties. While the ARC does not disaggregate its wheat breeding budget by wheat type, an explanation for this variance could be that a larger portion of the breeding budget is allocated to winter wheat improvements.

### Producer benefits

Producer benefits, measured as revenue gains attributable to the ARC’s wheat breeding program from 1992 to 2015, are presented in Table 4. Using the cumulative estimated yearly genetic gain increases, actual area sown to ARC varieties by type (spring, winter, and facultative) and price data from 1992 to 2015, we can roughly estimate the total revenue gain to South African wheat producers. Table 4 indicates that the total gains attributable to the ARC’s wheat breeding program were estimated to total $106,453,777 (2016 USD) or $4,435,574 (2016 USD) annually. These amounts are less than those estimated by [28], who found the ARC wheat breeding program added $22.6 million (2016 USD) annually from 1980 to 2008. The higher results estimated by [28] could be attributed to the time period; after 1997, there was a significant drop in South African wheat area planted due to market liberalization. As such, the study by [28] captured the benefits of many more wheat hectares and substantially higher wheat prices. So, the genetic gain could be the same, but due to exogenous factors like hectares sown to ARC lines and wheat price, the gains estimated by [28] would be higher. Second, estimates from [28] were not derived from empirical yield data but rather by analyzing macro-level data. Thus, the estimates of this study, which are calculated from empirical yield data, provide results that are field driven and not aggregated to the macro level, unlike those found in [28].

**Table 4:** Total gains attributable to Agricultural Research Council’s wheat breeding program: 1992–2015.

Year	ARC Spring Wheat 2016 USD Gain^a^	ARC Winter Wheat 2016 USD Gain^a^	ARC Facultative Wheat 2016 USD Gain^a^	Total Gain
1992	-	$0	-	$0
1993	-	$889,883	-	$889,883
1994	$0	$1,370,726	$0	$1,370,726
1995	$6,196,165	$4,393,759	$2,995,895	$13,585,820
1996	$7,383,586	$2,971,035	$5,732,029	$16,086,650
1997	$4,317,121	$2,911,336	$4,413,257	$11,641,714
1998	$609,714	$1,371,870	$2,715,039	$4,696,623
1999	$1,120,027	$750,030	$485,003	$2,355,060
2000	$298,592	$180,052	$162,262	$640,906
2001	$894,941	$1,268,584	$879,891	$3,043,416
2002	$511,713	$1,221,789	$981,796	$2,715,298
2003	$774,929	$111,935	$834,471	$1,721,335
2004	$1,004,965	$193,635	$1,129,278	$2,327,879
2005	$1,228,832	$587,387	$2,391,127	$4,207,346
2006	$3,729,061	$683,236	$1,195,980	$5,608,277
2007	$4,416,923	$2,545,220	$674,475	$7,636,618
2008	$3,326,080	$1,610,870	$2,190,503	$7,127,452
2009	$2,860,107	$301,101	$492,057	$3,653,265
2010	$2,869,010	$642,019	$1,937,771	$5,448,800
2011	$4,013,537	$472,868	$651,407	$5,137,812
2012	$2,840,696	$395,680	$314,776	$3,551,151
2013	$1,414,313	$232,144	$79,943	$1,726,401
2014	$463,165	$224,542	$38,523	$726,230
2015	$55,691	$380,520	$118,904	$555,115
			Average	**$4,435,574**
			Total	**$106,453,777**

^a^ As derived from 2016 prices from Table 4 and cumulative genetic gain used on Table 3

Another way of interpreting our benefit estimates is a counterfactual scenario. That is, what would have happened if the ARC had not invested in wheat breeding from 1992 to 2014? The implicit counterfactual is that South African producers would have continued to grow varieties of the vintage and yield of Tugela-DN and forfeited the benefits estimated above.

However, it is more likely South African producers would have adopted wheat varieties developed by other breeding programs (CIMMYT, Pannar, and Sensako, for example). In that sense, the estimates derived above would overestimate the total benefits of the ARC breeding program. Considered from another point of view, this study likely underestimates the true benefits of the ARC wheat breeding program, because it does not include the contribution made by ARC in terms of pathogen/disease/pest resistance. Globally, producers tend to focus on yield potential (ceilings) of varieties instead of variability (floors) and, thus, may often undervalue the genetic resistance to a disease that does not raise yield potential but raises the yield floor. More explicitly, when ARC breeds for Russian wheat aphid resistance, for example, it does not raise the yield potential of a given variety because Russian wheat aphids are not present every growing season, and yield potential is derived from a best-case scenario. However, disease/pest/pathogen resistance does, in fact, reduce the yield variability (floor) of a variety. Economists and policymakers tend to undervalue the opportunity cost of this type of agricultural research, specifically with regard to maintenance (pathogen/disease/pest resistance) breeding. Furthermore, maintenance breeding for biotic and abiotic stresses stabilize yields overtime which reduces price variability which benefits poor consumers. Accordingly, the substantial economic benefit that accrues from avoided yield losses through resistance to pathogens is often omitted in the cost-benefit analysis of such breeding programs because the producers do not experience the losses, but breeding programs incur the costs to prevent them. While we do not explicitly estimate the benefits of resistance, we implicitly acknowledge their important contribution.

### Benefit-cost analysis

ARC provided breeding costs for their wheat breeding program (Table 5). These costs include all breeding, pre-breeding, laboratory, salaries, and other expenses associated with the program. Ideally, costs would be distributed over the 10 years prior to release, representing the time it takes to initially cross a variety and its eventual commercial release. However, ARC wheat breeding cost data were only available from 2004 to 2015, so lagging was not an option. We linearly extrapolate costs from 2004 to 1992 and adjust for inflation, with the assumption the breeding program began in 1991. While these extrapolations are synthesized data, they do provide plausible estimates of investment return. We estimate (Table 5) the benefit-cost ratio [41] to be 5.1:1 as of 1991. That is, for every dollar invested in the ARC wheat breeding program, a return of $5.10 is generated. The benefit-cost ratio provides evidence that the economic rate of return to the ARC wheat breeding program is high, although evaluating these measures further is difficult without comparable values for other public investments (the opportunity cost of public funds).

**Table 5:** Cost-benefit analysis of the ARC wheat breeding program: 1992–2015.

Year^a^	Costs (2016 USD)	Benefits (2016 USD)^b^
1992	$1,204,967	$0
1993	$1,157,053	$889,883
1994	$1,107,952	$1,370,726
1995	$1,055,208	$13,585,820
1996	$1,031,498	$16,086,650
1997	$982,730	$11,641,714
1998	$981,071	$4,696,623
1999	$935,746	$2,355,060
2000	$941,295	$640,906
2001	$909,867	$3,043,416
2002	$894,494	$2,715,298
2003	$827,780	$1,721,335
2004	$756,428	$2,327,879
2005	$820,042	$4,207,346
2006	$844,848	$5,608,277
2007	$841,515	$7,636,618
2008	$863,566	$7,127,452
2009	$848,179	$3,653,265
2010	$690,641	$5,448,800
2011	$689,671	$5,137,812
2012	$605,516	$3,551,151
2013	$600,437	$1,726,401
2014	$658,406	$726,230
2015	$634,556	$555,115
	**$20,883,468**	**$106,453,777**
	**BCR**	**5.10**

^a^Actual costs provided by ARC were used from 2004 to 2015 and costs from 1992 to 2003 were linearly extrapolated.

^b^Using data from Table 4, we calculate total benefits by the following equation:

$\sum_{t=1}^{t}Ait\;\Upsilon it\;Pt$, where A_it_ is area of ARC wheat type i in year t (1992–2015), *Υ*_it_ is cumulative genetic gain for ARC wheat type i in year t, and P_t_ is wheat price in 2016 USD in year t. The benefit-cost ratio (BCR) is calculated as a measure of gross research benefits: $\frac{\sum_{t}\frac{B_{t}}{{\left(1+r\right)}^{t}}}{\sum_{t}\frac{C_{t}}{{\left(1+r\right)}^{t}}}$, where B_t_ is the total economic benefit in year t, C_t_ represents annual program costs, and r is the assumed discount rate of 10.25%.

To put this in context, [5] found the mean benefit-cost ratio for agricultural investment in Sub-Saharan Africa to be 30:1. The 5.1:1 ratio would be in the second quartile of 129 benefit-cost ratio studies applied to Africa. Our relatively low ratio could be due to a litany of factors including the fact that ARC/SGI uses its funds for a multitude of activities besides wheat breeding. ARC conducts holistic research for the public good in areas including wheat breeding, soil cultivation, pest and disease control, and farmer training, which are all incorporated into its wheat breeding budget. Furthermore, adherence by the ARC to the relatively high wheat quality standards in the country may be a major cause of the low returns from ARC wheat breeding, when measured through yield gains. ARC must breed for higher yield and higher quality, which are inversely related. However, taking from the estimates in this research, the benefits of the ARC-SGI’s wheat breeding program outweigh the costs $5.1 to $1, demonstrating that investments in the ARC breeding program have provided large and sustained economic benefits to wheat producers in South Africa.

### Consumer benefits

Impacts associated with R & D investments are broad and include productivity gains, increased incomes, nutritional benefits and changes in household resource allocation patterns. South Africa wheat consumers benefit from increased supply and the subsequently reduced domestic price. While modeling the price effects of the increased supply attributed to the ARC is outside the scope of this study, we are able to estimate the increased number of wheat rations annually attributed to the ARC. Table 6 indicates that on average the ARC wheat breeding program provides an additional 253,318 wheat rations annually, assuming a per capita consumption of 60.9 kg/yr [12]. We also measured changes in consumer and producer surplus attributable to the ARC wheat breeding [42, 43]. We assumed a 0.51% improvement in yields due to wheat breeding (found as the percentage of total yields attributed to the ARC wheat breeding program (Table 3) to the total South African wheat supply). As is typical, consumers gained more in consumer surplus than producers lost in producer surplus. The net surplus summed over 1992–2015 was $42.64 million (Table 6). This compares with the cost of the ARC wheat breeding program of $21 million. Consumer surplus can be reflective of the benefits to consumers of lower prices emanating from increased quantity supplied to markets emanating from agricultural research and development. These benefits can enhance food security and other socio-economic imperatives.

**Table 6:** Changes in producer and consumer surplus and additional wheat rations attributed to the ARC wheat breeding program: 1992–2015.

Year	Additional kg of wheat^a^	Additional rations^b^	Consumer Surplus (2016 USD)^c^	Producer Surplus (2016 USD)^c^	Net Surplus (2016 USD)
1992	0	0	0	0	0
1993	2,328,744	38,239	2,803,240	-906,548	1,896,692
1994	3,978,799	65,333	2,105,106	-680,777	1,424,329
1995	39,427,738	647,418	2,494,100	-806,574	1,687,525
1996	49,803,101	817,785	3,128,942	-1,011,878	2,117,064
1997	44,045,147	723,237	1,907,222	-616,782	1,290,439
1998	21,817,882	358,258	1,354,479	-438,029	916,450
1999	10,392,658	170,651	1,358,722	-439,401	919,321
2000	2,738,711	44,971	2,053,384	-664,050	1,389,334
2001	13,593,902	223,217	1,754,424	-567,368	1,187,055
2002	13,649,861	224,136	1,794,807	-580,428	1,214,379
2003	6,989,524	114,771	1,651,072	-533,945	1,117,127
2004	10,847,291	178,116	1,874,785	-606,292	1,268,492
2005	21,055,668	345,742	1,990,724	-643,786	1,346,938
2006	20,928,157	343,648	2,670,781	-863,712	1,807,069
2007	18,547,217	304,552	4,118,357	-1,331,848	2,786,509
2008	22,892,110	375,897	3,836,274	-1,240,624	2,595,649
2009	17,205,658	282,523	2,395,391	-774,653	1,620,738
2010	15,661,346	257,165	3,277,351	-1,059,873	2,217,479
2011	14,752,913	242,248	4,850,069	-1,568,479	3,281,591
2012	9,571,851	157,173	4,943,625	-1,598,734	3,344,891
2013	5,617,366	92,239	3,762,574	-1,216,790	2,545,784
2014	2,546,731	41,818	3,876,338	-1,253,581	2,622,758
2015	1,856,882	30,491	3,015,039	-975,042	2,039,996
	Average	**253,318**	**2,739,861**	**-886,052**	**1,853,809**
	Total	**6,079,627**	**63,016,804**	**-20,379,196**	**42,637,609**

^a^Summation of cumulative genetic gain kg/ha for each wheat type on Table 3.

^b^Assuming a per capital wheat consumption of 60.9 kg annually [12].

^c^ The elasticity of demand was set at -0.22, the elasticity of supply at 0.68 and the upward shift in supply at 0.51% assuming demand held constant. Prices and quantities varied with the year observed.

## Conclusions

Although classified as an upper middle-income country by the World Bank, food insecurity is still a concern throughout South Africa. Food insecurity was prevalent between 2014 and 2015 in South Africa when a severe drought left 22% of South African households food insecure [3]. This reinforced food security as a prominent political and agricultural concern. Food security relates to a number of factors including increased and consistent yields of modern staple crop varieties. South Africa become a net wheat importer beginning in 1990 leaving it vulnerable to the volatility of international wheat price and global exchange rates. Domestic and international factors have led to the South African rand dropping significantly against the USD (by 58% between 2012 and 2017 [10]). This implies importing wheat to ensure food security will be more costly and more volatile.

One way the South African government has combated food insecurity is by funding the South African Agricultural Research Council’s (ARC) Small Grains Institute (SGI) to improve crop yields with new wheat varieties. Importantly, unlike some other regional studies [17, 18, 39], we find that South African genetic gains in wheat have not plateaued. Thus, further yield increases could be expected from additional research. We also find that, except for winter wheat, newer varieties have increased yields while not increasing the variance of wheat yields, implying a decreasing coefficient of variation.

The high quality standards set by the research technical committee (RTC) of the South African wheat industry could be undermining potential yield increases in South Africa. These standards encourage millers to import lower quality wheat from abroad. Quality attributes were not available for this study, so yield losses due to quality standards cannot be measured. That said, the presence of strict wheat quality standards combined with the fact that one of the general characteristics of wheat is the defect of conversion (yield declines as quality improves) may help to explain why the genetic gains modeled in this study have been increasing over time at a decreasing rate.

This study likely underestimates the benefits of ARC genetic material if ARC materials were used by private wheat breeders in South Africa over the study period. The advent of plant variety protection (PVP) and intellectual property rights (IPR) laws considerably restricted the exchange of plant genetic resources and breeding lines between the public and private wheat breeding research institutions in South Africa. The ARC contributed to some of the plant breeders’ rights owned by private companies through shared genetic resources made available before the PVP/IPRs were implemented [44]. Without knowing how much, if any, of the genetic material being used by private breeders in South Africa originated with the ARC, it is not feasible to estimate the additional benefits this study neglects. Thus, it is likely that the estimates of benefits in this study could be the lower bound if ARC genetic material was used in private breeding over the study period.

This study is only one part of a larger effort to develop sustainable wheat production to ensure global food security. Achieving this goal in the face of increased wheat demand and climate change requires integrated approaches across plant breeding, economic, agronomic, soil, biologic, hydrologic, and other scientific disciplines whose research can be influenced by the results provided in this study. Similarly, the ARC wheat breeding program is one part of a holistic effort to ensure food security in South Africa and neighboring countries which import wheat from South Africa. Continued funding for public plant breeding programs such as ARC-SGI would likely lead to wheat breeders raising the yield ceiling and ensuring that maintenance breeding for disease and pest resistance simultaneously raises the yield floor. In addition, our benefit-cost analysis suggests ample returns to ARC funding. Feeding a growing population will need to be met with both increased domestic supply supplemented with global imports. Increasing domestic supply through public and private wheat breeding, extension services, and holistic scientific agricultural research will help shield South Africa from an uncertain global grain market and risk from exchange rate fluctuations. Continued funding for public plant breeding programs such as the ARC wheat breeding program ensures that genetic gains accomplished by wheat breeders globally avoid plateauing, which is a potent tool for combating global food insecurity. Food security will not flow from plant breeding alone. This study shows the payoff from investment in enhanced breeding is one piece of the puzzle of achieving food security.

## Supporting information

S1 Fig South African actual wheat yields vs Agricultural Research Council’s experimental test plot wheat yield average observations: 1998–2014. (TIF)

S2 Fig Yield observations by normal and late planting: 1998–2014. (TIF)

S3 Fig Yield observations by irrigated and dryland production: 1998–2008. (TIF)

S4 Fig Yield observations by variety type: 1998–2014. (TIF)

S5 Fig Yield observations by growing year. (TIF)

S6 Fig Yield observations by release year and varietal type. (TIF)

S1 Table Percent of total South African wheat planted to ARC cultivars: 1992–2015. (DOCX)

S2 Table Planting and harvesting rules used by Agricultural Research Council’s test plot locations across South Africa. (DOCX)

S3 Table Percent. of Agricultural Research Council’s total wheat area planted by wheat type: 1992–2015. (DOCX)

S4 Table Fixed effects regression results from the OLS and Just-Pope models for spring irrigated wheat varieties. (DOCX)

S5 Table Fixed effects regression results from OLS and Just-Pope production models for spring dryland wheat varieties. (DOCX)

S6 Table Fixed effects regression results from OLS and Just-Pope models for winter wheat varieties. (DOCX)

S7 Table Fixed effects regression results from OLS and Just-Pope models for facultative wheat varieties. (DOCX)

S8 Table South African Agricultural Research Council average yield by wheat type: 1998–2014. (DOCX)

S9 Table Yearly average yield for All ARC test plots. (DOCX)

S10 Table Average yield of ARC varieties by the year it was commercially released (RLYR). (DOCX)

S11 Table Average ARC test plot yields by plot and location: 1998–2014. (DOCX)
