# Supplementary figures and images for: The role of public wheat breeding in reducing food insecurity in South Africa
Source: PLoS One. 2018 Dec 31;13(12):e0209598. doi: 10.1371/journal.pone.0209598 (PMC6312393; doi:10.1371/journal.pone.0209598)

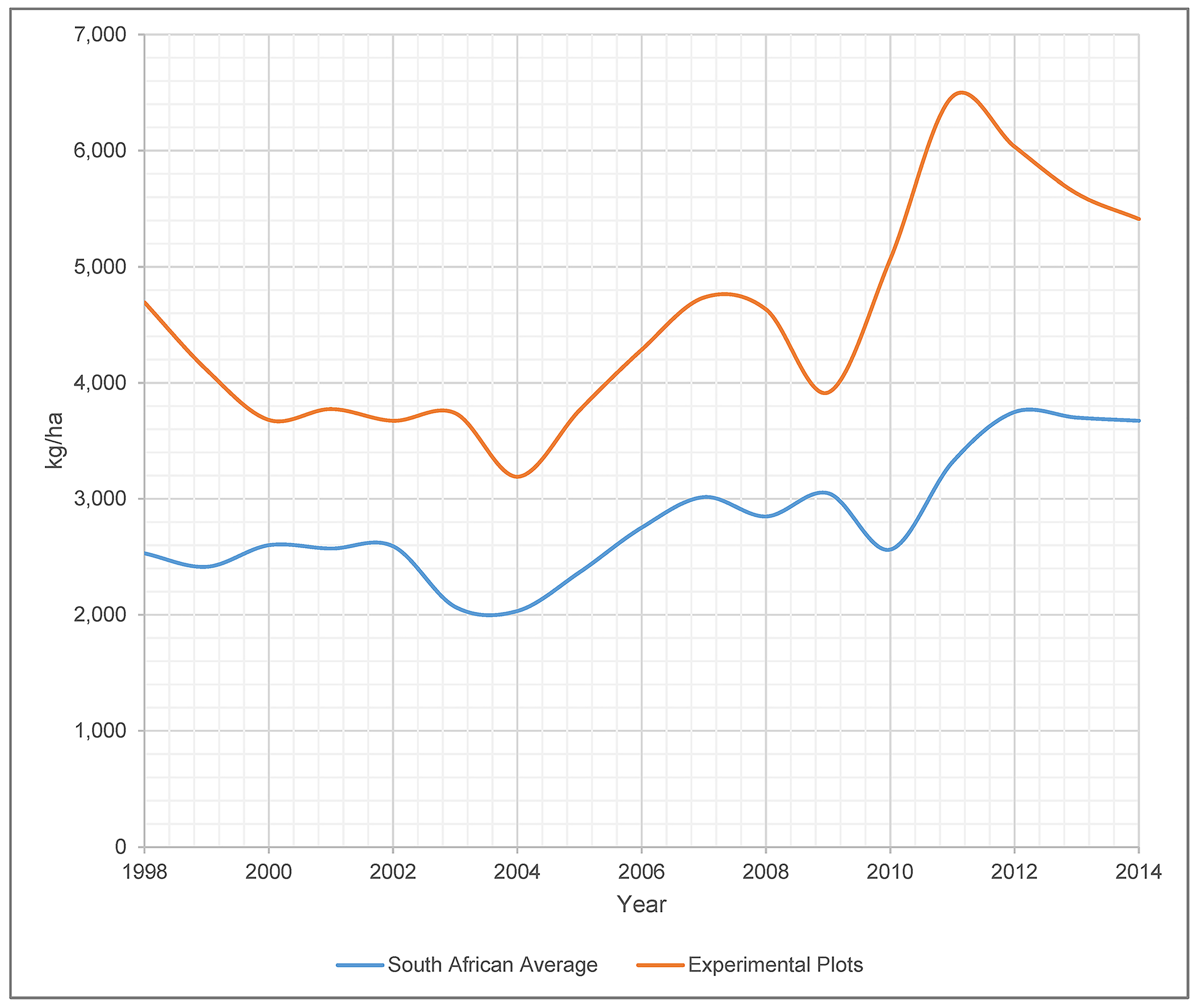

Supplement: S1 Fig — (TIF) [file pone.0209598.s001.tif]

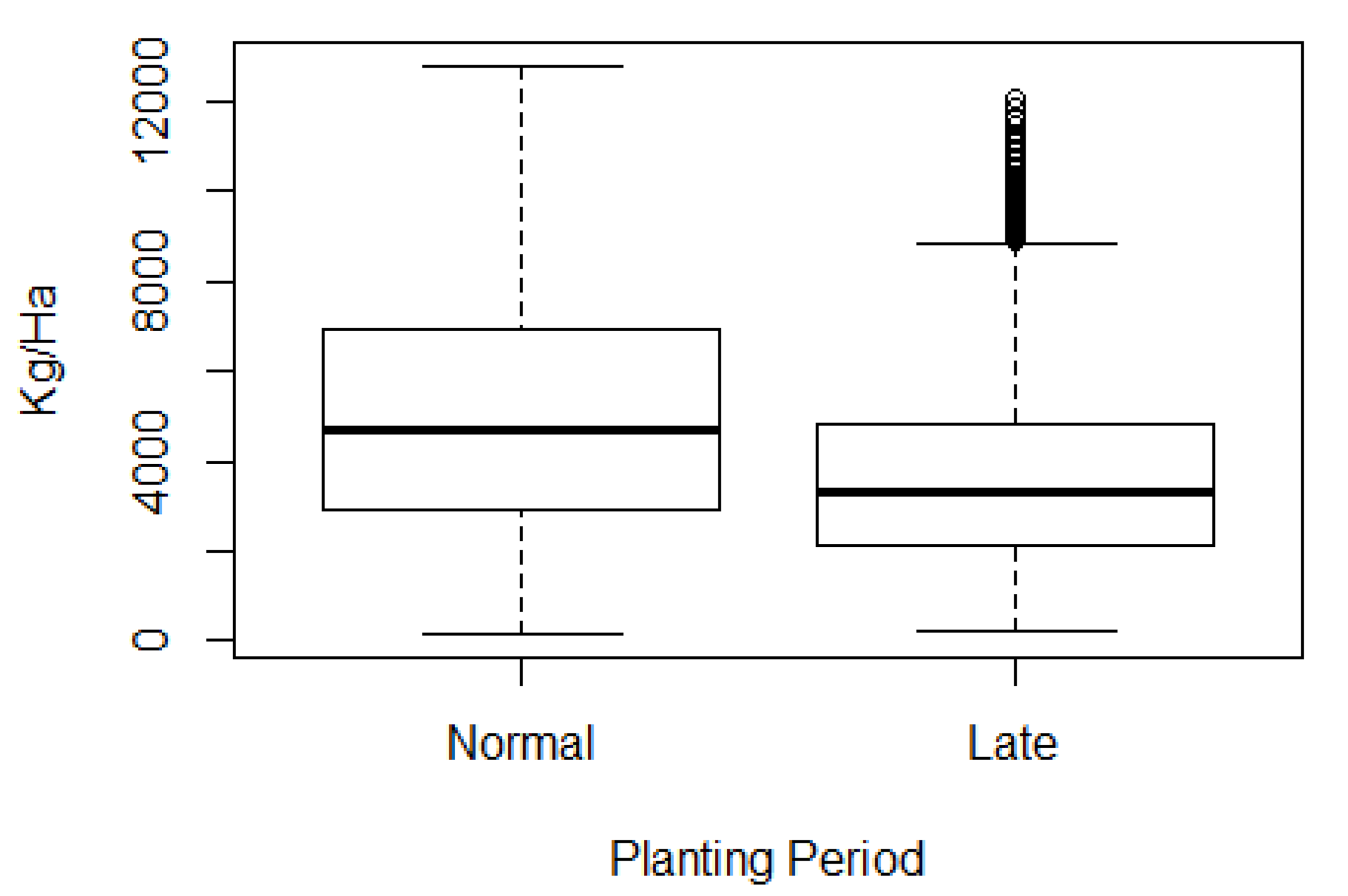

Supplement: S2 Fig — (TIF) [file pone.0209598.s002.tif]

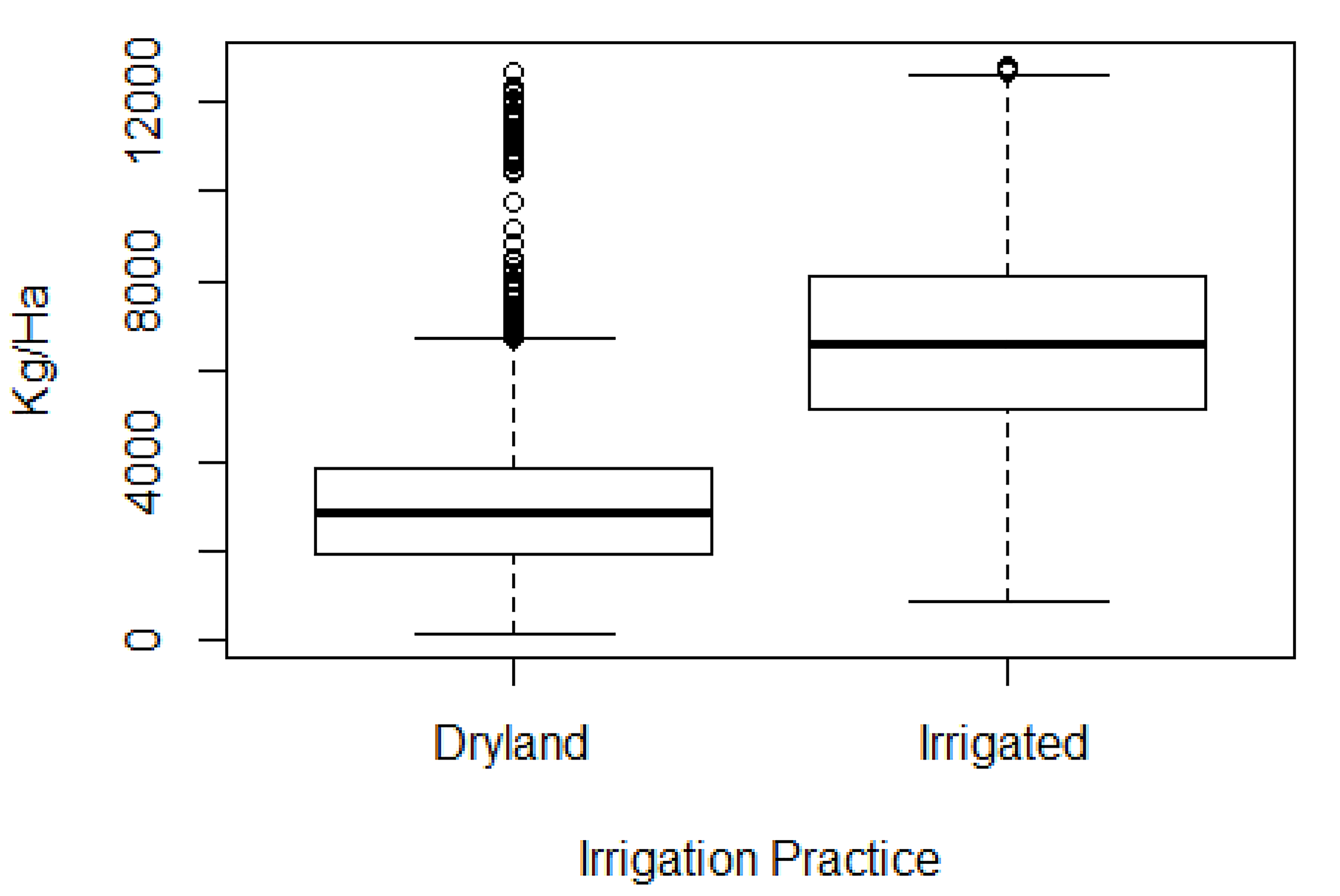

Supplement: S3 Fig — (TIF) [file pone.0209598.s003.tif]

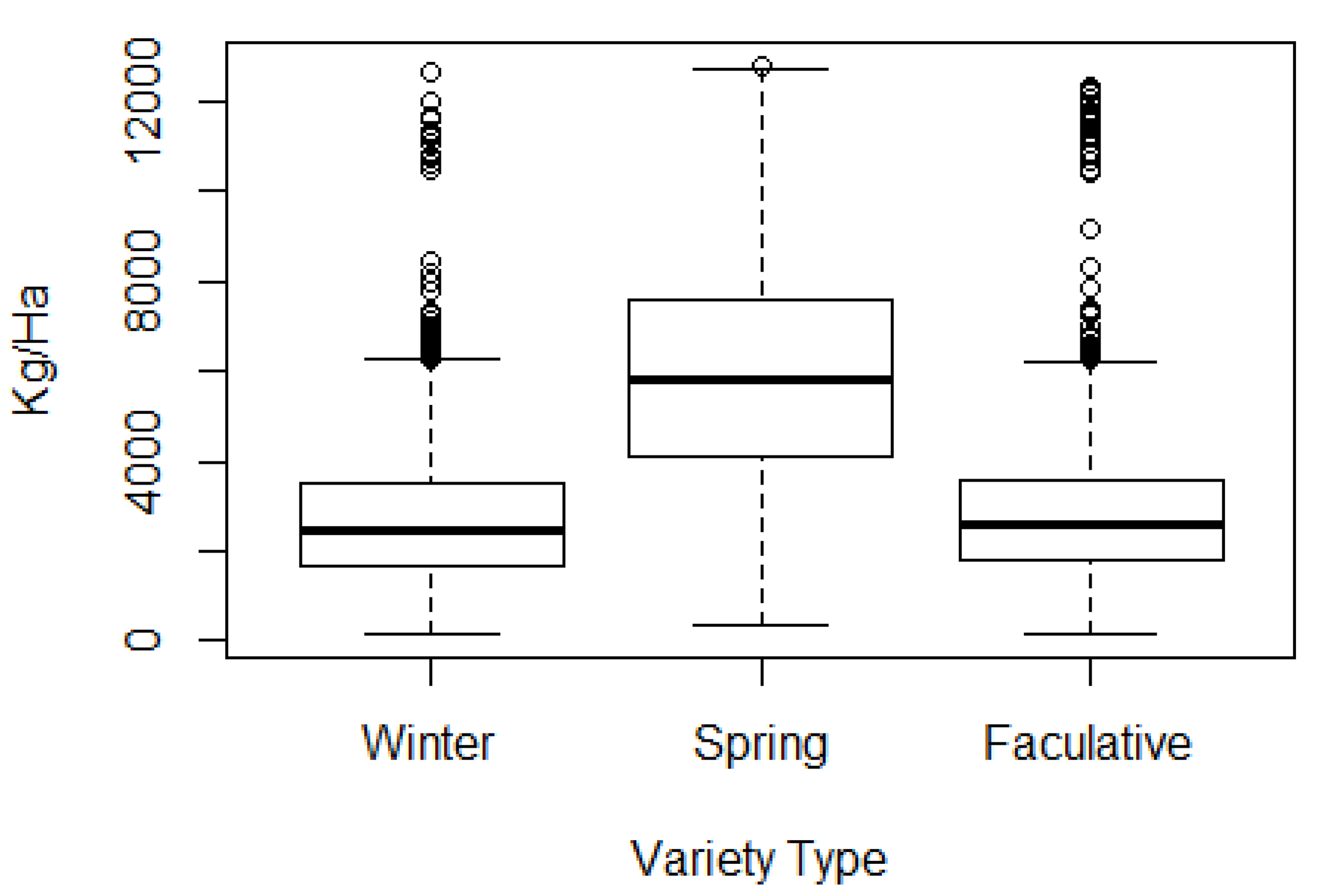

Supplement: S4 Fig — (TIF) [file pone.0209598.s004.tif]

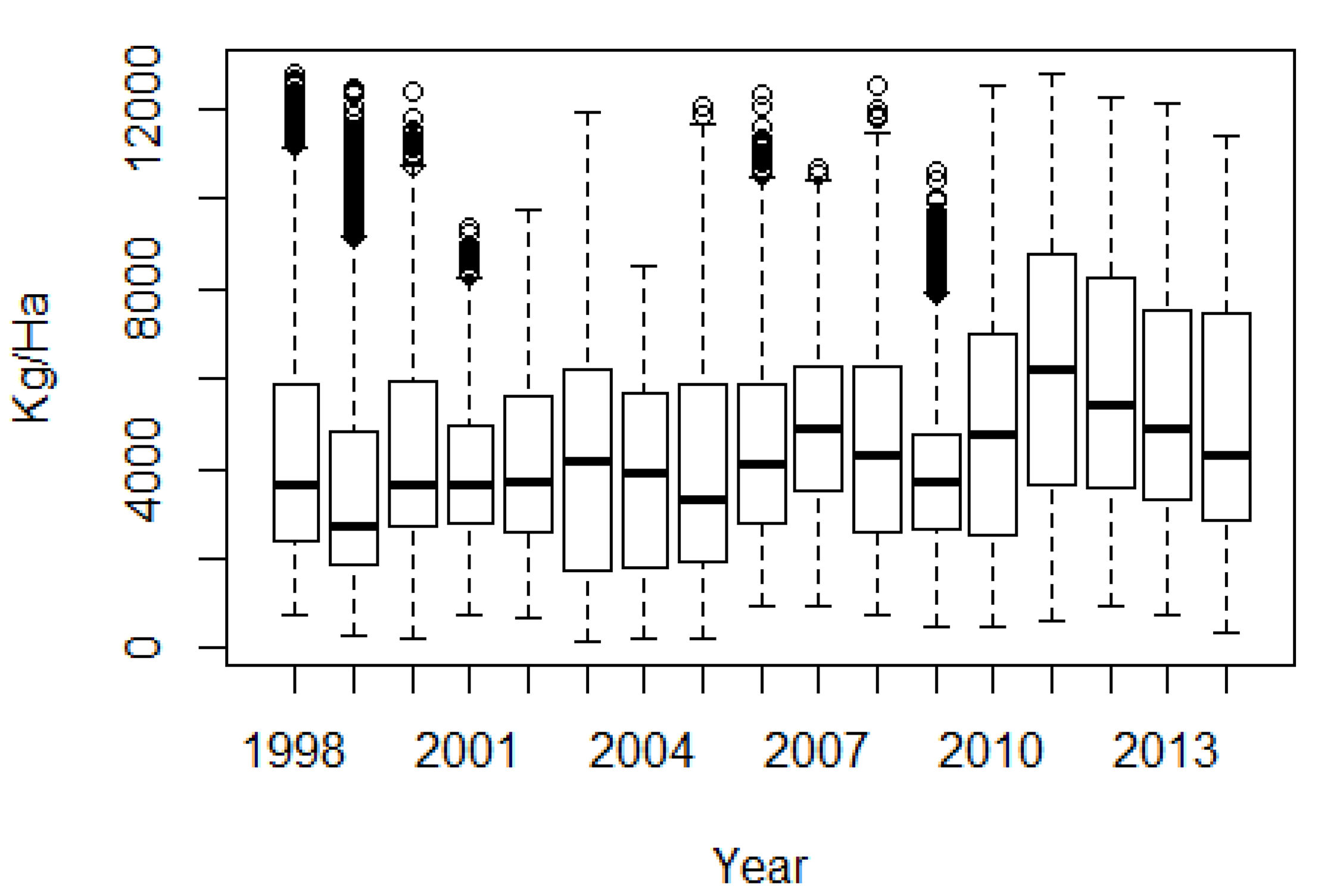

Supplement: S5 Fig — (TIF) [file pone.0209598.s005.tif]

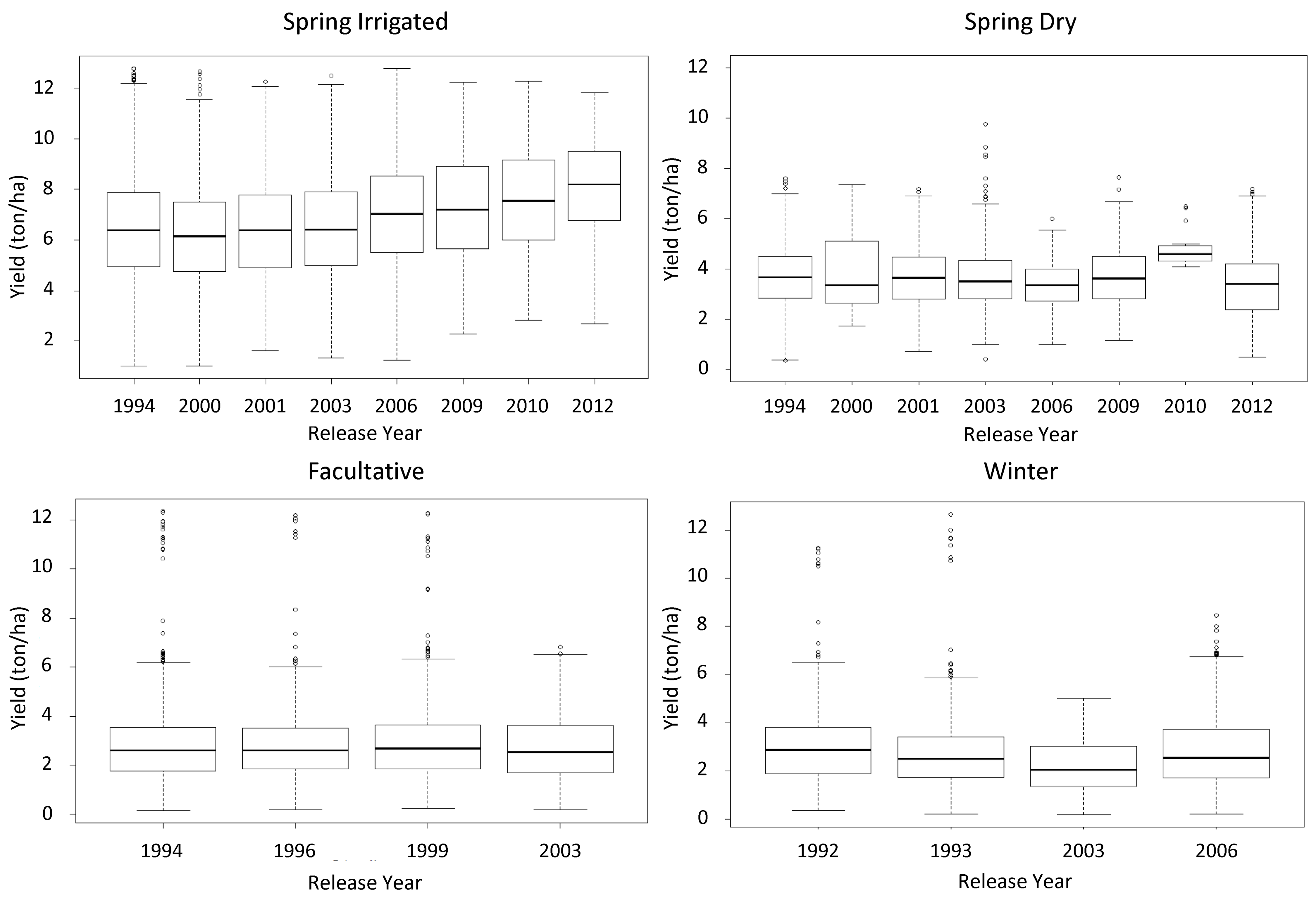

Supplement: S6 Fig — (TIF) [file pone.0209598.s006.tif]
