# Supplementary material for: The role of public wheat breeding in reducing food insecurity in South Africa
Source: PLoS One. 2018 Dec 31;13(12):e0209598. doi: 10.1371/journal.pone.0209598 (PMC6312393; doi:10.1371/journal.pone.0209598)
Supplement: S1 Table — (DOCX) [file pone.0209598.s007.docx]

**S1 Table. Percent of Total South African Wheat Planted to ARC Cultivars: 1992–2015**

| Year | % of Total Winter | % of Total Spring | % of Total Facultative | ARC Spring Wheat (Ha) | ARC Winter Wheat (Ha) | ARC Facultative Wheat (Ha) |
| --- | --- | --- | --- | --- | --- | --- |
|  |  |  |  |  |  |  |
| 1992 | 62.26 | 63.64 | 12.39 | 425,083 | 6,631 | 8,066 |
| 1993 | 96.35 | 60.85 | 72.53 | 468,428 | 20,612 | 200,382 |
| 1994 | 100.00 | 64.80 | 76.89 | 533,572 | 22,218 | 148,430 |
| 1995 | 100.00 | 52.10 | 72.25 | 508,235 | 56,428 | 238,609 |
| 1996 | 100.00 | 56.89 | 57.01 | 407,621 | 35,062 | 307,282 |
| 1997 | 90.21 | 33.75 | 35.01 | 230,790 | 37,713 | 229113 |
| 1998 | 80.42 | 10.60 | 37.56 | 34,566 | 20,091 | 149,071 |
| 1999 | 58.62 | 8.99 | 22.67 | 54,040 | 9,765 | 22,723 |
| 2000 | 69.70 | 1.84 | 2.91 | 12,846 | 2,148 | 6,779 |
| 2001 | 77.89 | 5.30 | 14.73 | 37,658 | 15,097 | 35,954 |
| 2002 | 74.22 | 3.20 | 20.84 | 22,936 | 15,714 | 42,731 |
| 2003 | 23.08 | 4.24 | 25.05 | 26,771 | 1,122 | 27,994 |
| 2004 | 49.06 | 5.55 | 30.65 | 38,257 | 2,158 | 41,746 |
| 2005 | 58.95 | 7.72 | 55.43 | 48,480 | 6,833 | 91,607 |
| 2006 | 59.45 | 15.64 | 43.66 | 106,282 | 5,776 | 33,099 |
| 2007 | 93.69 | 13.71 | 32.38 | 79,629 | 13,678 | 11,808 |
| 2008 | 54.07 | 12.28 | 51.78 | 77,277 | 11,203 | 49,421 |
| 2009 | 51.48 | 13.13 | 52.43 | 74,963 | 3,010 | 15,900 |
| 2010 | 36.45 | 11.90 | 49.16 | 56,100 | 3,845 | 37,396 |
| 2011 | 20.08 | 14.32 | 27.18 | 78,107 | 2,781 | 12,310 |
| 2012 | 28.61 | 10.43 | 36.24 | 50,942 | 2,149 | 5,482 |
| 2013 | 18.89 | 6.14 | 23.34 | 30,094 | 1,499 | 1,652 |
| 2014 | 36.32 | 2.23 | 23.00 | 10,450 | 1,541 | 844 |
| 2015 | 19.54 | 0.26 | 18.75 | 1,181 | 2,457 | 2,448 |
