# Supplementary material for: The role of public wheat breeding in reducing food insecurity in South Africa
Source: PLoS One. 2018 Dec 31;13(12):e0209598. doi: 10.1371/journal.pone.0209598 (PMC6312393; doi:10.1371/journal.pone.0209598)
Supplement: S2 Table — (DOCX) [file pone.0209598.s008.docx]

**S2 Table. Planting and Harvesting Rules used by Agricultural Research Council’s Test Plot Locations Across South Africa**

| Production Type | Planting Period | Region | Planting | Emergence | Harvest |
| --- | --- | --- | --- | --- | --- |
| Irrigated | Early | All | Site Specific | 7 days after planting | 30 days after flowering |
| Irrigated | Late | All except  Eastern Highveld | 2 weeks after  planting period 1 | 7 days after planting | 30 days after flowering |
| Irrigated | Late | Eastern Highveld | 2 weeks after  planting period 1 | 7 days after planting | 37 days after flowering |
| Dryland | Early | All | Site Specific | 10 days after planting | 30 days after flowering |
| Dryland | Late | Ruens and Swartland | 2 weeks after  planting period 1 | 10 days after planting | 30 days after flowering |
| Dryland | Late | NWFS, SWFS, CFS | 3 weeks after  planting period 1 | 10 days after planting | 30 days after flowering |
| Dryland | Late | Eastern Free State | 4 weeks after planting period 1 | 10 days after planting | 30 days after flowering |
