# Supplementary material for: The role of public wheat breeding in reducing food insecurity in South Africa
Source: PLoS One. 2018 Dec 31;13(12):e0209598. doi: 10.1371/journal.pone.0209598 (PMC6312393; doi:10.1371/journal.pone.0209598)
Supplement: S3 Table — (DOCX) [file pone.0209598.s009.docx]

**S3 Table. Percent of Agricultural Research Council’s Total Wheat Area Planted by Wheat Type: 1992–2015**

|  | % of Area | | | | % of Wheat Type | | |
| --- | --- | --- | --- | --- | --- | --- | --- |
| Year | ARC | Winter | Spring | Facultative | ARC Spring Wheat | ARC Winter Wheat | ARC Facultative Wheat |
| 1992 | 58.64 | 1.42 | 89.06 | 8.68 | 63.64 | 62.26 | 12.39 |
| 1993 | 64.21 | 1.99 | 71.61 | 25.70 | 60.85 | 96.35 | 72.53 |
| 1994 | 67.21 | 2.12 | 78.57 | 18.42 | 64.80 | 100.00 | 76.89 |
| 1995 | 59.00 | 4.14 | 71.57 | 24.23 | 52.10 | 100.00 | 72.25 |
| 1996 | 58.17 | 2.71 | 55.38 | 41.66 | 56.89 | 100.00 | 57.01 |
| 1997* | 42.68 | 3.03 | 49.49 | 47.36 | 33.75 | 90.21 | 35.01 |
| 1998 | 27.18 | 3.34 | 43.60 | 53.06 | 10.60 | 80.42 | 37.56 |
| 1999 | 8.96 | 2.32 | 83.72 | 13.96 | 8.99 | 58.62 | 22.67 |
| 2000 | 2.27 | 0.33 | 74.75 | 24.94 | 1.84 | 69.70 | 2.91 |
| 2001 | 8.43 | 1.99 | 72.95 | 25.06 | 5.30 | 77.89 | 14.73 |
| 2002 | 8.65 | 2.25 | 76.17 | 21.79 | 3.20 | 74.22 | 20.84 |
| 2003 | 7.24 | 0.65 | 84.41 | 14.94 | 4.24 | 23.08 | 25.05 |
| 2004 | 9.90 | 0.53 | 83.05 | 16.41 | 5.55 | 49.06 | 30.65 |
| 2005 | 18.23 | 1.44 | 78.01 | 20.53 | 7.72 | 58.95 | 55.43 |
| 2006 | 18.98 | 1.27 | 88.83 | 9.91 | 15.64 | 59.45 | 43.66 |
| 2007 | 16.64 | 2.31 | 91.90 | 5.77 | 13.71 | 93.69 | 32.38 |
| 2008 | 18.44 | 2.77 | 84.13 | 12.76 | 12.28 | 54.07 | 51.78 |
| 2009 | 17.51 | 0.91 | 88.86 | 4.72 | 13.13 | 51.48 | 52.43 |
| 2010 | 17.44 | 1.89 | 84.47 | 13.63 | 11.90 | 36.45 | 49.16 |
| 2011 | 15.41 | 2.29 | 90.20 | 7.49 | 14.32 | 20.08 | 27.18 |
| 2012 | 11.46 | 1.47 | 98.58 | 2.96 | 10.43 | 28.61 | 36.24 |
| 2013 | 6.67 | 1.57 | 96.96 | 1.40 | 6.14 | 18.89 | 23.34 |
| 2014 | 2.69 | 0.89 | 98.33 | 0.77 | 2.23 | 36.32 | 23.00 |
| 2015 | 1.29 | 2.62 | 94.62 | 2.72 | 0.26 | 19.54 | 18.75 |

*1997 does not exist due to report transition from Wheat Board to South African Grain Laboratories. Values for 1997 were estimated by averaging 1996 and 1998 values.
