# Supplementary material for: The role of public wheat breeding in reducing food insecurity in South Africa
Source: PLoS One. 2018 Dec 31;13(12):e0209598. doi: 10.1371/journal.pone.0209598 (PMC6312393; doi:10.1371/journal.pone.0209598)
Supplement: S4 Table — (DOCX) [file pone.0209598.s010.docx]

**S4 Table. Fixed Effects Regression Results from the OLS and Just-Pope Models for Spring Irrigated Wheat Varieties**

| Parameter | OLS Yield | Just-Pope Variance | Just-Pope Yield |
| --- | --- | --- | --- |
| Intercept | 6643.89 [147.06]*** | 14.45 [0.22]*** | 6671.36 [433.97]*** |
| AMERSFOORT | 169.86 [241.52] | -1.00 [0.36]** | 174.52 [565.07] |
| ATLANTA | 287.51 [130.79]* | 0.01 [0.19] | 294.03 [668.18] |
| BARKLEY_WEST | 1417.21 [93.91]*** | 0.19 [0.14] | 1419.96 [520.21]** |
| BEDFORD | 1447.03 [374.68]*** | -2.02 [0.55]** | 1449.96 [349.32]*** |
| BERGVILLE | -65.50 [114.67] | -0.39 [0.17]* | -56.41 [432.81] |
| BLOEMHOF | 1877.52 [209.83]*** | 0.19 [0.31] | 1908.41 [789.97]* |
| BLOUDRIFT | 639.32 [378.98] | -1.17 [0.56]* | 649.50 [389.44] |
| BRITS | 355.36 [98.01]** | -0.61 [0.15]*** | 354.66 [395.61] |
| BULLHILL | 1981.48 [94.74]*** | -0.07 [0.14] | 1983.23 [395.04]*** |
| BULTFONTEIN | 796.35 [284.64]** | 1.57 [0.42]** | 782.68 [1255.06] |
| BURGERSFORT | 183.61 [201.05] | -0.67 [0.30]* | 192.95 [578.22] |
| CHRISTIANA | 2318.69 [125.17]*** | -0.59 [0.19]** | 2323.47 [419.81]*** |
| CLARENS | 2524.65 [528.84]*** | -3.61 [0.78]*** | 2533.66 [478.85]*** |
| DANIELSRUS | 1377.29 [437.40]** | -3.05 [0.65]*** | 1395.94 [414.69]** |
| DELMAS | 2695.57 [430.33]*** | -1.44 [0.64]* | 2697.41 [347.44]*** |
| DOUGLAS | 2244.40 [111.40]*** | -0.21 [0.16] | 2255.58 [422.23]*** |
| DUNDEE | -530.65 [158.41]** | -0.22 [0.23] | -515.93 [614.08] |

| FRANKFORT | 1816.69 [215.59]*** | -0.73 [0.32]* | 1834.46 [598.33]** |
| --- | --- | --- | --- |
| GREYTOWN | -912.97 [374.68]* | -1.75 [0.55]** | -910.12 [349.32]* |
| GROBLERSDAL | 604.75 [134.91]*** | -0.85 [0.20]*** | 615.22 [440.53] |
| HARRISMITH | 927.12 [528.84] | -5.37 [0.78]*** | 936.07 [478.85] |
| HARTSWATER | 3822.48 [129.96]*** | -0.77 [0.19]*** | 3823.59 [489.30]*** |
| HOOPSTAD | 2201.27 [159.95]*** | 0.10 [0.24] | 2200.75 [573.92]** |
| HOPETOWN | 2631.71 [102.93]*** | -0.23 [0.15] | 2637.78 [492.99]*** |
| KANONEILAND | 3496.62 [206.69]*** | -0.48 [0.31] | 3496.93 [371.49]*** |
| KOEDOESKOP | 1000.18 [120.94]*** | -1.15 [0.18]*** | 997.55 [393.67]* |
| LADYBRAND | 1319.87 [149.16]*** | -0.13 [0.22] | 1326.33 [501.18]* |
| LICHTENBURG | 1450.21 [117.20]*** | -0.63 [0.17]** | 1450.36 [472.35]** |
| LOSKOP | 1754.25 [139.07]*** | -0.79 [0.21]*** | 1757.81 [419.30]*** |
| MAGALIESBURG | 478.53 [186.12]* | -0.35 [0.28] | 457.92 [715.74] |
| MAKOPPA | 1175.39 [340.13]** | -0.95 [0.50] | 1208.46 [666.28] |
| MARBLEHALL | -149.90 [160.68] | -1.19 [0.24]*** | -140.20 [405.38] |
| MODDERFONTEIN | 2345.60 [268.79]*** | -1.44 [0.40]** | 2345.91 [437.15]*** |
| MODDERIVIER | -493.04 [146.78]** | -0.01 [0.22] | -489.74 [522.46] |
| NABOOMSPRUIT | 719.85 [125.66]*** | -0.48 [0.19]** | 726.44 [450.70] |
| NEWCASTLE | -786.23 [243.01]** | -1.49 [0.36]*** | -782.47 [422.97] |
| NYLSTROOM | -495.09 [308.68] | -1.61 [0.46]** | -489.41 [317.91] |
| OHRIGSTAD | 646.91 [154.85]*** | -0.19 [0.23] | 654.76 [564.95] |
| ORANJEVILLE | 2350.08 [285.77]*** | -2.49 [0.42]*** | 2366.53 [524.65]*** |
| PLOOYSBURG | 1817.69 [430.87]*** | -3.26 [0.64]*** | 1807.50 [332.18]*** |
| POTCHEFSTROOM | 364.52 [157.39]* | -0.15 [0.23] | 364.47 [581.79] |
| PRIESKA | 2174.06 [99.21]*** | -0.10 [0.15] | 2181.21 [417.29]*** |
| RAMA | 2364.42 [92.94]*** | -0.41 [0.14]** | 2364.23 [344.19]*** |
| REITZ | 2342.06 [386.39]*** | -0.79 [0.57] | 2330.64 [575.85]** |
| REMHOOGTE | 2562.62 [99.72]*** | 0.18 [0.15] | 2573.33 [411.27]*** |
| RIETRIVER | 2409.41 [98.33]*** | 0.55 [0.15]** | 2415.11 [594.16]** |
| SANDVET | 2166.40 [430.33]*** | -2.06 [0.64]** | 2167.99 [347.44]*** |
| SKUINDRIFT | 1365.65 [166.51]*** | 0.21 [0.25] | 1378.33 [796.41] |
| STANDERTON | 699.39 [201.44]** | -0.88 [0.30]** | 694.97 [726.60] |
| TAUNG | -915.91 [254.69]** | -0.55 [0.38] | -915.80 [447.22]* |
| THEUNISSEN | 831.19 [145.62]*** | -0.99 [0.22]*** | 842.63 [273.54]** |
| UPINGTON | 1949.77 [99.82]*** | -0.56 [0.15]*** | 1949.57 [499.47]** |
| VAALHARTS | 2296.93 [83.27]*** | -0.34 [0.12]** | 2301.01 [492.88]*** |
| VAALWATER | -44.75 [164.00] | -0.07 [0.24] | -33.68 [478.73] |
| VERENA | 2187.24 [430.33]*** | -1.17 [0.64] | 2188.30 [347.44]*** |
| VILLIERS | 1018.81 [153.03]*** | -0.73 [0.23]** | 1034.31 [472.71]* |
| VRYHEID | -1463.78 [307.66]*** | -1.57 [0.46]** | -1465.92 [450.91]** |
| WINTERTON | -503.62 [135.72]** | -0.50 [0.20]* | -492.69 [513.88] |
| 1999 | -434.56 [175.18]* | 0.07 [0.26] | -473.06 [400.64] |
| 2000 | -2029.68 [155.81]*** | -0.63 [0.23]** | -2067.42 [262.75]*** |
| 2001 | -3132.53 [163.27]*** | -0.68 [0.24]** | -3160.88 [327.46]*** |
| 2002 | -2007.25 [157.08]*** | -1.20 [0.23]*** | -2034.39 [267.49]*** |
| 2003 | -2172.03 [146.18]*** | -0.60 [0.22]** | -2200.32 [231.98]*** |
| 2004 | -2499.19 [146.53]*** | -0.80 [0.22]** | -2546.24 [404.08]*** |
| 2005 | -1268.21 [153.71]*** | -0.76 [0.23]** | -1284.81 [265.50]*** |
| 2006 | -1335.89 [146.23]*** | -0.90 [0.22]*** | -1365.78 [307.07]*** |
| 2007 | -1558.18 [147.89]*** | -0.87 [0.22]*** | -1581.88 [271.15]*** |
| 2008 | -1622.01 [155.32]*** | -0.88 [0.23]*** | -1658.88 [288.68]*** |
| 2009 | -2369.62 [162.42]*** | -1.12 [0.24]*** | -2406.55 [296.19]*** |
| 2010 | -1329.54 [155.40]*** | -1.21 [0.23]*** | -1363.06 [258.63]*** |
| 2011 | -269.65 [150.27] | -0.90 [0.22]*** | -297.31 [258.96] |
| 2012 | -323.52 [156.37]* | -0.91 [0.23]*** | -355.50 [257.78] |
| 2013 | -931.86 [175.05]*** | -0.90 [0.26]** | -977.26 [324.47]** |
| 2014 | -699.37 [173.12]*** | -0.31 [0.26] | -735.09 [419.56] |
| Late planting | -482.01 [48.93]*** | -0.26 [0.07]** | -483.40 [168.98]** |
| logrlyr | 65.13 [22.02]*** | 0.02 [0.03] | 64.81 [29.37]*** |
| R^2^ | 0.4935 | 0.0625 | 0.4896 |
| P value for Year | <0.0001 | <0.0001 | <0.0001 |
| P value for Station | <0.0001 | <0.0001 | <0.0001 |
| Number of Clusters | - | - | 51 |
| Mean Yield (kg/ha) | 6686.59 | - | 6696.4 |
| Nobs | 8,527 | 8,527 | 8,527 |

*** (P<0.01), ** (P<0.05), *(P<0.10)
