# Supplementary material for: The role of public wheat breeding in reducing food insecurity in South Africa
Source: PLoS One. 2018 Dec 31;13(12):e0209598. doi: 10.1371/journal.pone.0209598 (PMC6312393; doi:10.1371/journal.pone.0209598)
Supplement: S5 Table — (DOCX) [file pone.0209598.s011.docx]

**S5 Table. Fixed Effects Regression Results from OLS and Just-Pope Production Models for Spring Dryland Wheat Varieties**

| Parameter | OLS Yield | Just-Pope Variance | Just-Pope Yield |
| --- | --- | --- | --- |
| Intercept | 2756.02 [174.58]*** | 12.25 [0.43]*** | 2725.48 [346.00]*** |
| ADOWA | -836.00 [455.61] | -3.76 [1.13]** | -818.87 [318.34]* |
| ALPHA | 1355.25 [167.97]*** | 0.09 [0.42] | 1380.40 [433.17]** |
| ARLINGTON | -1052.02 [182.88]*** | -2.09 [0.45]*** | -1040.85 [322.41]** |
| BOONTJIESKRAAL | 1946.48 [460.88]*** | -1.26 [1.14] | 1977.02 [346.00]*** |
| BOTHAVILLE | 920.96 [184.11]*** | 0.22 [0.46] | 928.67 [344.73]* |
| BREDASDORP | 271.37 [172.82] | -1.00 [0.43]* | 295.99 [335.76] |
| BULTFONTEIN | 575.86 [176.02]** | 0.30 [0.44] | 591.11 [521.60] |
| CALEDON | 814.79 [282.38]** | -0.44 [0.70] | 827.44 [264.12]** |
| CLARENS | 1212.56 [185.40]*** | 2.70 [0.46]*** | 1206.18 [850.02] |
| CLOCOLAN | -861.51 [217.22]*** | -1.37 [0.54]* | -849.30 [209.30]** |
| DEVLEI | 534.00 [455.61] | -2.52 [1.13]* | 551.13 [318.34] |
| EENDEKUIL | 1388.35 [183.01]*** | -0.32 [0.45] | 1415.99 [417.15]** |
| ELSENBURG | 3703.61 [336.74]*** | -0.68 [0.84] | 3727.50 [401.33]*** |
| EXCELSIOR | -1747.72 [260.80]*** | -3.57 [0.65]*** | -1725.13 [268.94]*** |
| FICKSBURG | 466.92 [178.12]** | -0.02 [0.44] | 472.52 [352.99] |
| GELUKSFONTEIN | -1340.98 [329.31]*** | -3.68 [0.82]*** | -1336.90 [225.78]*** |
| HALFMANSHOF | 625.09 [161.59]*** | 0.47 [0.40] | 631.40 [403.59] |
| HARRISMITH | 1189.79 [188.59]*** | 2.08 [0.47]*** | 1175.98 [619.67] |
| HEBRON | -1304.95 [220.62]*** | -3.04 [0.55]*** | -1286.96 [326.80]** |
| HEIDELBERG | 752.87 [253.01]** | -0.23 [0.63] | 763.69 [372.04] |
| HOPEFIELD | 643.48 [159.40]*** | -0.46 [0.40] | 660.73 [358.86] |
| KLEINFONTEIN | -278.19 [183.01] | 0.28 [0.45] | -268.38 [459.26] |
| KLIPDALE | 976.71 [164.58]*** | -1.21 [0.41]** | 995.52 [343.36]** |
| KOPERFONTEIN | 257.94 [188.55] | -0.68 [0.47] | 270.20 [311.57] |
| KORINGBERG | 738.95 [187.35]*** | -0.11 [0.46] | 768.27 [406.99] |
| LADYBRAND | -625.84 [174.13]** | -0.75 [0.43] | -621.23 [142.71]** |
| LANGGEWENS | 1503.51 [159.44]*** | 0.25 [0.40] | 1532.17 [394.24]** |
| LANGRUG | 2163.20 [186.39]*** | 0.15 [0.46] | 2184.90 [444.39]*** |
| MALMESBURY | 2111.28 [170.38]*** | -0.06 [0.42] | 2133.55 [401.09]*** |
| MEADOWS | -1473.90 [195.21]*** | -2.34 [0.48]*** | -1468.84 [292.55]*** |
| MOORREESBURG | 1965.48 [161.75]*** | -0.22 [0.40] | 1990.24 [337.10]*** |
| NAPIER | 442.27 [172.79]* | 0.58 [0.43] | 454.99 [468.97] |
| PHILADELPHIA | 2437.09 [168.83]*** | 0.03 [0.42] | 2461.50 [356.96]*** |
| PIKETBERG | 996.41 [170.38]*** | -0.71 [0.42] | 1023.72 [363.43]* |
| POOLS | 1000.21 [160.67]*** | 1.02 [0.40]* | 1021.07 [665.76] |
| PORTERVILLE | 1089.01 [168.67]*** | -0.36 [0.42] | 1111.66 [418.64]* |
| PROTEM | 830.61 [164.76]*** | -0.41 [0.41] | 838.25 [418.72] |
| REITZ | 104.31 [185.50] | 1.25 [0.46]** | 104.38 [267.05] |
| RIETPOEL | 2533.98 [460.88]*** | -2.02 [1.14] | 2564.52 [346.00]*** |
| RIVERSDAL | 1464.07 [168.44]*** | 0.11 [0.42] | 1475.82 [402.62]** |
| RIVIERSONDEREND | 1843.51 [282.38]*** | -1.51 [0.70]* | 1852.10 [264.12]*** |
| RONNEPLEEGTE | -1294.32 [188.39]*** | -2.49 [0.47]*** | -1285.81 [277.76]*** |
| ROODEBLOEM | 1840.46 [166.60]*** | 0.03 [0.41] | 1852.13 [249.02]*** |
| SAMESUING | -682.40 [348.19] | -1.52 [0.86] | -650.69 [308.32]* |
| SERJANTSRIVIER | 2443.98 [460.88]*** | -0.34 [1.14] | 2474.52 [346.00]*** |
| SWELLENDAM | 1076.64 [162.68]*** | 0.11 [0.40] | 1088.08 [537.86] |
| TWEESPRUIT | -1100.87 [197.10]*** | -0.98 [0.49]* | -1097.53 [454.36]* |
| TYGERHOEK | 1433.42 [171.05]*** | -0.21 [0.42] | 1454.09 [406.92]** |
| UITVLUG | 1875.19 [180.31]*** | 0.50 [0.45] | 1910.29 [484.87]** |
| VELDDRIFT | 1320.78 [218.23]*** | -1.10 [0.54]* | 1322.32 [300.18]** |
| VOORSTEKOP | 678.56 [179.34]** | -1.16 [0.45]** | 691.98 [383.62] |
| VREDENBURG | 141.57 [245.71] | -1.48 [0.61]* | 162.26 [415.16] |
| WESSELSBRON | 198.40 [189.99] | -0.03 [0.47] | 207.99 [547.00] |
| WINBURG | -1373.67 [329.00]*** | -3.23 [0.82]*** | -1341.96 [308.32]** |
| WITSAND | 437.40 [192.55]* | 0.07 [0.48] | 447.16 [580.84] |
| 2008 | -272.52 [143.84] | 0.48 [0.36] | -259.12 [123.47]* |
| 2009 | -380.64 [138.82]** | 0.49 [0.34] | -368.96 [118.29]** |
| 2010 | -1158.26 [137.29]*** | -0.24 [0.34] | -1138.12 [112.04]*** |
| 2011 | -114.54 [125.40] | 0.09 [0.31] | -114.29 [168.95] |
| 2012 | 256.52 [126.09]* | -0.05 [0.31] | 293.16 [358.40] |
| 2013 | 29.35 [127.57] | 0.54 [0.32] | 29.58 [252.30] |
| 2014 | -456.05 [131.38]** | 0.00 [0.33] | -434.90 [226.64] |
| late_planting 1 | 293.19 [71.77]*** | -0.37 [0.18]* | 309.84 [104.17]** |
| logrlyr | 20.82 [36.23] | -0.12 [0.09] | 19.79 [44.73] |
| R^2^ | 0.5513 | 0.206301 | 0.521 |
| P value for Year | <0.0001 | <0.0001 | <0.0001 |
| P value for Station | <0.0001 | <0.0001 | <0.0001 |
| Number of Clusters | - | - | 22 |
| Mean Yield (kg/ha) | 3501.9 | - | 3548.5 |
| Nobs | 2,299 | 2,299 | 2,299 |

*** (P<0.01), ** (P<0.05), *(P<0.10)
