# Supplementary material for: The role of public wheat breeding in reducing food insecurity in South Africa
Source: PLoS One. 2018 Dec 31;13(12):e0209598. doi: 10.1371/journal.pone.0209598 (PMC6312393; doi:10.1371/journal.pone.0209598)
Supplement: S6 Table — (DOCX) [file pone.0209598.s012.docx]

**S6 Table. Fixed Effects Regression Results from OLS and Just-Pope Models for Winter Wheat Varieties**

| Parameter | OLS | JP VAR | JP Yield |
| --- | --- | --- | --- |
| Intercept | 3765.50 [82.43]*** | 12.65 [0.19]*** | 3821.29 [251.55]*** |
| AMERSFOORT | 339.19 [254.24] | 0.15 [0.57] | 338.35 [129.25]* |
| ARLINGTON | -928.62 [82.93]*** | -0.88 [0.19]*** | -917.07 [110.09]*** |
| BLOEMFONTEIN | -1604.02 [154.88]*** | 0.27 [0.35] | -1610.97 [372.18]** |
| BOTHAVILLE | -100.24 [104.69] | 0.23 [0.24] | -121.45 [297.81] |
| BULTFONTEIN | 694.42 [71.30]*** | 0.78 [0.16]*** | 677.15 [377.23] |
| CLARENS | 928.30 [80.73]*** | 0.01 [0.18] | 925.27 [132.34]*** |
| CLOCOLAN | 71.46 [93.27] | 0.94 [0.21]*** | 68.14 [340.51] |
| EXCELSIOR | -630.61 [86.43]*** | 0.42 [0.19]* | -624.57 [489.32] |
| FICKSBURG | 778.38 [85.73]*** | 1.11 [0.19]*** | 792.40 [251.66]** |
| FRANKFORT | 538.99 [146.87]** | 2.64 [0.33]*** | 600.38 [967.59] |
| GELUKSFONTEIN | -1778.28 [267.52]*** | -1.36 [0.60]* | -1766.29 [179.89]*** |
| HARRISMITH | 485.89 [90.29]*** | -0.09 [0.20] | 485.86 [217.58]* |
| HEBRON | -1226.93 [76.91]*** | 0.25 [0.17] | -1244.93 [273.78]*** |
| HENNENMAN | -679.77 [98.26]*** | -0.58 [0.22]** | -696.54 [230.34]** |
| KROONSTAD | -2186.29 [349.70]*** | -3.52 [0.79]*** | -2242.07 [252.75]*** |
| LADYBRAND | 90.59 [75.77] | 0.45 [0.17]** | 94.66 [197.55] |
| MEADOWS | -1484.73 [178.29]*** | -1.50 [0.40]** | -1483.94 [173.36]*** |
| PETRUSBURG | -1289.93 [80.10]*** | -0.44 [0.18]* | -1304.87 [267.91]*** |
| PETRUSSTEYN | -744.75 [129.09]*** | 0.01 [0.29] | -800.77 [438.61] |
| REITZ | -278.26 [86.32]** | 0.34 [0.19] | -305.56 [268.86] |
| RONNEPLEEGTE | -1545.55 [212.33]*** | -1.27 [0.48]** | -1549.98 [272.51]*** |
| SAMESUING | -575.76 [156.78]** | -0.17 [0.35] | -586.02 [167.54]** |
| SENEKAL | -962.04 [113.20]*** | -0.53 [0.26]* | -998.11 [314.84]** |
| TWEESPRUIT | -910.23 [85.57]*** | -0.28 [0.19] | -937.95 [336.92]** |
| WESSELSBRON | 419.77 [78.80]*** | 0.45 [0.18]* | 423.78 [191.90]* |
| WINBURG | -1482.40 [495.13]** | -3.20 [1.12]** | -1487.82 [234.43]*** |
| 1999 | -1476.15 [87.87]*** | -1.10 [0.20]*** | -1522.34 [230.96]*** |
| 2000 | -776.86 [98.25]*** | -1.26 [0.22]*** | -828.07 [264.68]** |
| 2001 | -482.30 [103.51]*** | -1.32 [0.23]*** | -551.17 [306.79] |
| 2002 | -1360.95 [88.63]*** | -2.08 [0.20]*** | -1420.19 [261.28]*** |
| 2003 | -2211.72 [91.46]*** | -1.15 [0.21]*** | -2263.28 [238.72]*** |
| 2004 | -2131.18 [84.53]*** | -1.15 [0.19]*** | -2199.39 [268.40]*** |
| 2005 | -1898.94 [81.54]*** | -1.38 [0.18]*** | -1952.84 [218.94]*** |
| 2006 | -398.11 [78.14]*** | -1.22 [0.18]*** | -438.41 [227.53] |
| 2007 | -39.99 [83.07] | 0.16 [0.19] | -88.26 [221.57] |
| 2008 | -1849.35 [108.13]*** | -0.65 [0.24]** | -1912.47 [257.15]*** |
| 2009 | -653.08 [84.38]*** | -1.05 [0.19]*** | -694.51 [258.79]* |
| 2010 | -1705.19 [106.19]*** | -0.76 [0.24]** | -1771.27 [279.49]*** |
| 2011 | -1339.06 [129.93]*** | -1.18 [0.29]*** | -1394.88 [327.86]** |
| 2012 | -235.58 [115.56]* | -0.72 [0.26]** | -287.24 [270.31] |
| 2013 | -899.68 [128.90]*** | -0.38 [0.29] | -926.98 [240.46]** |
| 2014 | -1127.13 [125.34]*** | -1.16 [0.28]*** | -1196.92 [292.20]** |
| late_planting | 8.68 [30.34] | 0.23 [0.07]** | 2.84 [64.35] |
| logrlyr | 160.99 [16.35]*** | 0.12 [0.04]** | 163.00 [50.39]** |
| R^2^ | 0.5547 | 0.127566 | 0.5455 |
| P value for Year | 0.0001 | 0.0001 | 0.0001 |
| P value for Station | 0.0001 | 0.0001 | 0.0001 |
| Number of Clusters | - | - | 29 |
| Mean Yield (kg/ha) | 2719.72 | - | 2760.2 |
| Nobs | 4287 | 4287 | 4287 |

*** (P<0.01), ** (P<0.05), *(P<0.10)
