# Supplementary material for: The role of public wheat breeding in reducing food insecurity in South Africa
Source: PLoS One. 2018 Dec 31;13(12):e0209598. doi: 10.1371/journal.pone.0209598 (PMC6312393; doi:10.1371/journal.pone.0209598)
Supplement: S7 Table — (DOCX) [file pone.0209598.s013.docx]

**S7 Table. Fixed Effects Regression Results from OLS and Just-Pope Models for Facultative Wheat Varieties**

| Parameter | OLS | JP VAR | JP Yield |
| --- | --- | --- | --- |
| Intercept | 3749.42 [52.39]*** | 12.98 [0.12]*** | 3801.98 [306.65]*** |
| AMERSFOORT | 483.52 [152.67]** | -0.40 [0.36] | 484.99 [106.59]*** |
| ARLINGTON | -1135.60 [49.29]*** | -0.90 [0.12]*** | -1119.85 [138.99]*** |
| BLOEMFONTEIN | -1419.73 [91.88]*** | -0.15 [0.22] | -1417.49 [217.59]*** |
| BOTHAVILLE | -100.65 [63.35] | -0.11 [0.15] | -99.90 [212.39] |
| BULTFONTEIN | 505.04 [42.32]*** | 0.53 [0.10]*** | 478.31 [357.62] |
| CLARENS | 795.88 [45.51]*** | -0.03 [0.11] | 793.86 [151.12]*** |
| CLOCOLAN | -276.47 [52.95]*** | 0.30 [0.12]* | -275.59 [296.36] |
| EXCELSIOR | -800.85 [52.56]*** | -0.05 [0.12] | -797.22 [461.92] |
| FICKSBURG | 771.45 [47.87]*** | 0.60 [0.11]*** | 784.04 [222.33]** |
| FRANKFORT | 425.89 [85.60]*** | 2.25 [0.20]*** | 486.75 [901.92] |
| GELUKSFONTEIN | -1360.89 [244.59]*** | -2.55 [0.58]*** | -1343.30 [150.00]*** |
| HARRISMITH | 401.67 [51.45]*** | 0.35 [0.12]** | 412.52 [245.52] |
| HEBRON | -1292.11 [45.39]*** | -0.14 [0.11] | -1307.53 [228.40]*** |
| HENNENMAN | -753.69 [59.30]*** | -0.79 [0.14]*** | -769.06 [192.99]** |
| KROONSTAD | -1900.04 [234.62]*** | -2.42 [0.55]*** | -1953.03 [308.55]*** |
| LADYBRAND | -209.67 [44.74]*** | 0.08 [0.11] | -209.21 [220.36] |
| MEADOWS | -1428.73 [122.41]*** | -1.88 [0.29]*** | -1429.23 [224.73]*** |
| PETRUSBURG | -1333.66 [47.25]*** | -0.45 [0.11]*** | -1348.00 [259.69]*** |
| PETRUSSTEYN | -849.55 [79.14]*** | -0.34 [0.19] | -895.17 [303.79]** |
| REITZ | -421.31 [49.92]*** | 0.14 [0.12] | -446.36 [213.32]* |
| RONNEPLEEGTE | -1334.00 [140.12]*** | -1.13 [0.33]** | -1343.78 [269.51]*** |
| SAMESUING | -941.76 [90.52]*** | -0.50 [0.21]* | -940.99 [217.31]** |
| SENEKAL | -1046.45 [67.63]*** | -0.49 [0.16]** | -1072.37 [263.41]** |
| TWEESPRUIT | -1061.75 [50.85]*** | -0.22 [0.12] | -1085.32 [292.74]** |
| WESSELSBRON | 169.09 [46.72]** | 0.36 [0.11]** | 170.54 [175.04] |
| WINBURG | -1363.61 [270.66]*** | -2.78 [0.64]*** | -1365.05 [162.40]*** |
| 1999 | -1334.24 [58.90]*** | -1.28 [0.14]*** | -1375.30 [291.13]*** |
| 2000 | -537.80 [55.00]*** | -0.71 [0.13]*** | -582.64 [317.84] |
| 2001 | -237.05 [57.45]*** | -1.02 [0.14]*** | -305.37 [350.06] |
| 2002 | -1044.78 [55.13]*** | -2.11 [0.13]*** | -1097.61 [291.31]** |
| 2003 | -1911.87 [56.58]*** | -1.20 [0.13]*** | -1965.88 [284.49]*** |
| 2004 | -1716.46 [55.01]*** | -0.81 [0.13]*** | -1782.49 [304.78]*** |
| 2005 | -1369.09 [51.66]*** | -1.12 [0.12]*** | -1414.14 [258.25]*** |
| 2006 | -259.63 [48.72]*** | -1.02 [0.11]*** | -301.18 [271.21] |
| 2007 | 101.89 [51.50]* | 0.37 [0.12]** | 55.82 [263.08] |
| 2008 | -1818.24 [65.07]*** | -0.64 [0.15]*** | -1872.56 [288.66]*** |
| 2009 | -494.11 [51.74]*** | -1.19 [0.12]*** | -534.94 [296.60] |
| 2010 | -1404.51 [62.95]*** | -0.74 [0.15]*** | -1467.83 [296.73]*** |
| 2011 | -1063.83 [65.86]*** | -0.91 [0.16]*** | -1115.56 [296.49]** |
| 2012 | -123.05 [76.52] | -0.33 [0.18] | -158.14 [291.23] |
| 2013 | -1251.70 [96.81]*** | -0.30 [0.23] | -1299.41 [290.90]*** |
| 2014 | -1427.49 [92.69]*** | -0.57 [0.22]** | -1496.68 [312.35]*** |
| late_planting | 172.58 [18.07]*** | -0.09 [0.04]* | 170.29 [57.13]** |
| logrlyr | 51.90 [9.99]*** | -0.01 [0.02] | 52.57 [17.95]** |
| R^2^ | 0.5413 | 0.115 | 0.5319 |
| P value for Year | 0.0001 | 0.0001 | 0.0001 |
| P value for Station | 0.0001 | 0.0001 | 0.0001 |
| Number of Clusters | - | - | 29 |
| Mean Yield (kg/ha) | 2787.6 | - | 2825 |
| Nobs | 10,577 | 10,577 | 10,577 |

*** (P<0.01), ** (P<0.05), *(P<0.10)
