# Supplementary material for: The role of public wheat breeding in reducing food insecurity in South Africa
Source: PLoS One. 2018 Dec 31;13(12):e0209598. doi: 10.1371/journal.pone.0209598 (PMC6312393; doi:10.1371/journal.pone.0209598)
Supplement: S8 Table — (DOCX) [file pone.0209598.s014.docx]

**S8 Table. South African Agricultural Research Council Average Yield by Wheat Type: 1998–2014**

| Type | Observations | Yield  (kg/ha) | Standard Deviation of Yield  (kg/ha) |
| --- | --- | --- | --- |
| Winter | 4,287 | 2,719.72 | 1,433.15 |
| Spring | 21,643 | 5,935.23 | 2,319.87 |
| Facultative | 10,577 | 2,787.58 | 1,348.64 |
