# Supplementary material for: The role of public wheat breeding in reducing food insecurity in South Africa
Source: PLoS One. 2018 Dec 31;13(12):e0209598. doi: 10.1371/journal.pone.0209598 (PMC6312393; doi:10.1371/journal.pone.0209598)
Supplement: S9 Table — (DOCX) [file pone.0209598.s015.docx]

**S9 Table. Yearly Average Yield for All ARC Test Plots**

| Year | Observations | Average Yield (kg/ha) | Standard Deviation of Yield (kg/ha) |
| --- | --- | --- | --- |
|  |  |  |  |
| 1998 | 1,117 | 4,589.01 | 2,970.04 |
| 1999 | 1,092 | 3,853.68 | 2,946.00 |
| 2000 | 1,595 | 4,374.24 | 2,228.87 |
| 2001 | 1,180 | 4,024.95 | 1,665.92 |
| 2002 | 2,016 | 4,099.29 | 1,891.12 |
| 2003 | 2,500 | 4,267.24 | 2,606.85 |
| 2004 | 2,472 | 3,823.46 | 2,172.64 |
| 2005 | 3,018 | 3,976.78 | 2,550.67 |
| 2006 | 3,759 | 4,597.61 | 2,212.16 |
| 2007 | 2,941 | 4,933.92 | 2,014.76 |
| 2008 | 1,986 | 4,581.02 | 2,397.60 |
| 2009 | 2,824 | 3,895.67 | 1,715.75 |
| 2010 | 2,173 | 4,878.19 | 2,583.16 |
| 2011 | 3,493 | 6,197.23 | 3,001.12 |
| 2012 | 2,225 | 5,842.62 | 2,702.00 |
| 2013 | 976 | 5,360.08 | 2,627.47 |
| 2014 | 1,140 | 5,072.55 | 2,726.39 |
