# Supplementary material for: The role of public wheat breeding in reducing food insecurity in South Africa
Source: PLoS One. 2018 Dec 31;13(12):e0209598. doi: 10.1371/journal.pone.0209598 (PMC6312393; doi:10.1371/journal.pone.0209598)
Supplement: S10 Table — (DOCX) [file pone.0209598.s016.docx]

**S10 Table. Average Yield of ARC Varieties by the Year it Was Commercially Released (RLYR)**

| Release Year | Observations | Average Yield (kg/ha) | Standard Deviation of Yield (kg/ha) |
| --- | --- | --- | --- |
| 1992 | 416 | 3,123.48 | 1,748.23 |
| 1993 | 2,070 | 2,632.29 | 1,299.82 |
| 1994 | 9,718 | 4,403.33 | 2,429.97 |
| 1996 | 2,176 | 2,779.71 | 1,298.22 |
| 1999 | 2,407 | 2,873.14 | 1,367.65 |
| 2000 | 2,273 | 6,102.36 | 1,956.85 |
| 2001 | 3,305 | 5,526.89 | 2,202.30 |
| 2003 | 4,492 | 4,702.34 | 2,495.83 |
| 2006 | 5,225 | 5,705.96 | 2,714.89 |
| 2009 | 1,674 | 5,470.31 | 2,495.98 |
| 2010 | 843 | 7,474.59 | 2,015.39 |
| 2012 | 1,908 | 4,488.40 | 2,433.52 |
