# Supplementary material for: The role of public wheat breeding in reducing food insecurity in South Africa
Source: PLoS One. 2018 Dec 31;13(12):e0209598. doi: 10.1371/journal.pone.0209598 (PMC6312393; doi:10.1371/journal.pone.0209598)
Supplement: S11 Table — (DOCX) [file pone.0209598.s017.docx]

### S11 Table . Average ARC Test Plot Yields by Station and Location: 1998–2014

| Station | Province | Observations | Average Yield (Kg/Ha) | Standard Deviation of Yield (kg/ha) | Irrigated | Lat | Long | Altitude  (meters) |
| --- | --- | --- | --- | --- | --- | --- | --- | --- |
| ADOWA | Western Cape | 16 | 1,375.00 | 245.76 | no | -34.083 | 20.967 | 84 |
| ALPHA | Western Cape | 220 | 3,669.86 | 1,047.09 | no | -34.269 | 20.081 | 283 |
| AMERSFOORT | Free State | 132 | 3,670.38 | 1,443.72 | both | -27.033 | 28.600 | 1493 |
| ARLINGTON | Free State | 829 | 1,863.67 | 959.78 | no | -28.163 | 28.295 | 1631 |
| ATLANTA | Western Cape | 300 | 5,297.56 | 1,419.60 | yes | -33.665 | 18.582 | 71 |
| AURORA | Western Cape | 24 | 3,315.00 | 1,234.07 | no | -33.665 | 18.582 | 71 |
| BARKLEY WEST | Northern Cape | 760 | 6,782.38 | 2,283.51 | both | -28.534 | 24.272 | 1004 |
| BEDFORD | North-West | 28 | 6,903.93 | 710.85 | yes | -25.591 | 27.769 | 1085 |
| BERGVILLE | Kwazulu-Natal | 424 | 5,320.47 | 1,856.79 | yes | -28.819 | 29.402 | 1244 |
| BETHLEHEM | Free State | 2,635 | 3,933.89 | 1,484.62 | both | -28.163 | 28.295 | 1631 |
| BLOEMFONTEIN | Free State | 168 | 1,604.74 | 517.69 | both | -28.950 | 26.350 | 1304 |
| BLOEMHOF | North-West | 108 | 7,322.86 | 1,589.84 | yes | -27.633 | 25.600 | 1231 |
| BLOUDRIFT | North-West | 32 | 4,960.63 | 731.40 | yes | -27.383 | 25.550 | 1309 |
| BOLAND | Western Cape | 24 | 4,666.67 | 1,619.89 | no | -33.654 | 18.883 | 151 |
| BOONTJIESKRAAL | Western Cape | 48 | 2,767.08 | 1,035.28 | no | -34.200 | 19.350 | 128 |
| BOTHAVILLE | North-West | 452 | 3,138.66 | 1,078.02 | both | -27.383 | 25.550 | 1309 |
| BREDASDORP | Western Cape | 196 | 3,365.90 | 977.52 | no | -34.533 | 20.050 | 44 |
| BRITS | North-West | 732 | 5,816.86 | 1,211.69 | yes | -25.591 | 27.769 | 1085 |
| BULLHILL | Northern Cape | 739 | 7,383.21 | 1,835.68 | yes | -27.958 | 24.840 | 1180 |
| BULTFONTEIN | Free State | 1,476 | 3,699.90 | 1,343.53 | both | -28.300 | 26.150 | 1326 |
| BURGERSFORT | Limpopo | 112 | 5,797.64 | 1,026.87 | yes | -24.683 | 30.333 | 793 |
| CALEDON | Western Cape | 12 | 3,134.00 | 565.24 | no | -34.233 | 19.417 | 244 |
| CHRISTIANA | North-West | 342 | 8,096.77 | 1,446.00 | yes | -27.917 | 25.167 | 1207 |
| CLARENS | Free State | 1,027 | 3,836.50 | 1,313.83 | both | -28.517 | 28.417 | 1809 |
| CLOCOLAN | Free State | 628 | 2,766.87 | 1,014.65 | both | -28.921 | 27.584 | 1602 |
| DANIELSRUS | Free State | 20 | 7,165.53 | 502.81 | yes | -27.800 | 28.433 | 1615 |
| DELMAS | Mpumalanga | 24 | 7,274.58 | 783.83 | yes | -26.149 | 28.701 | 1532 |
| DEVLEI | Free State | 16 | 3,340.00 | 484.13 | no | -28.833 | 26.783 | 1454 |
| DOUGLAS | Mpumalanga | 451 | 7,473.62 | 1,634.84 | yes | -26.467 | 29.933 | 1675 |
| DUNDEE | Kwazulu-Natal | 184 | 4,119.58 | 1,144.50 | yes | -28.137 | 30.315 | 1219 |
| DUNHYEPARK | Western Cape | 8 | 4,377.50 | 780.73 | no | -34.315 | 19.503 | 189 |
| EENDEKUIL | Western Cape | 124 | 3,516.13 | 929.11 | no | -32.683 | 18.883 | 150 |
| ELSENBURG | Western Cape | 24 | 5,636.82 | 883.95 | no | -33.842 | 18.839 | 227 |
| EXCELSIOR | Eastern Cape | 676 | 2,259.03 | 1,860.22 | no | -31.017 | 25.667 | 1606 |
| FAIRFIELD | Western Cape | 24 | 3,666.67 | 614.14 | no | -34.233 | 19.417 | 244 |
| FICKSBURG | Free State | 852 | 3,762.66 | 1,518.14 | no | -28.867 | 27.883 | 1585 |
| FRANKFORT | Mpumalanga | 286 | 5,037.15 | 3,301.67 | both | -25.033 | 30.883 | 1005 |
| GELUKSFONTEIN | Free State | 40 | 1,097.38 | 173.78 | no | -28.950 | 26.350 | 1304 |
| GREYTOWN | Kwazulu-Natal | 28 | 4,368.21 | 804.99 | yes | -29.083 | 30.604 | 1043 |
| GROBLERSDAL | Limpopo | 287 | 6,131.12 | 1,386.79 | yes | -25.174 | 29.355 | 948 |
| HALFMANSHOF | Western Cape | 139 | 3,175.53 | 1,067.94 | no | -33.154 | 18.673 | 199 |
| HARRISMITH | Free State | 715 | 3,795.99 | 1,467.29 | both | -28.313 | 29.116 | 1723 |
| HARTSWATER | Northern Cape | 316 | 9,473.14 | 1,531.49 | yes | -28.534 | 24.272 | 1004 |
| HEBRON | Free State | 1,082 | 1,760.24 | 662.24 | no | -28.950 | 26.350 | 1304 |
| HEIDELBERG | Western Cape | 24 | 2,355.91 | 1,091.08 | no | -34.083 | 20.967 | 84 |
| HENNENMAN | Free State | 466 | 2,254.74 | 662.20 | no | -28.389 | 27.587 | 1587 |
| HEUNINGKLOOF | Western Cape | 12 | 3,659.17 | 625.80 | no | -34.083 | 20.967 | 84 |
| HOOPSTAD | Free State | 184 | 8,089.34 | 1,393.45 | yes | -27.817 | 25.900 | 1262 |
| HOPEFIELD | Western Cape | 208 | 3,184.03 | 580.46 | no | -33.067 | 18.350 | 30 |
| HOPETOWN | Northern Cape | 565 | 7,866.39 | 1,853.02 | yes | -29.581 | 24.149 | 1135 |
| KANONEILAND | Northern Cape | 102 | 9,844.27 | 1,046.41 | yes | -28.635 | 21.095 | 766 |
| KLEINFONTEIN | Limpopo | 76 | 2,282.99 | 685.27 | no | -23.666 | 29.790 | 1210 |
| KLIPDALE | Western Cape | 116 | 3,333.64 | 771.57 | no | -34.269 | 20.081 | 283 |
| KOEDOESKOP | Limpopo | 383 | 6,055.21 | 1,455.49 | yes | -24.882 | 27.521 | 939 |
| KOPERFONTEIN | Western Cape | 62 | 2,719.55 | 648.79 | no | -33.100 | 18.417 | 45 |
| KORINGBERG | Western Cape | 76 | 3,288.51 | 757.14 | no | -33.017 | 18.683 | 130 |
| KROONSTAD | Free State | 24 | 1,802.92 | 382.08 | no | -27.667 | 27.167 | 1338 |
| LADYBRAND | Free State | 1,363 | 3,486.66 | 1,816.18 | both | -29.112 | 27.453 | 1581 |
| LANGGEWENS | Western Cape | 295 | 3,877.38 | 1,041.04 | no | -33.283 | 18.700 | 177 |
| LANGRUG | Western Cape | 71 | 4,589.95 | 762.40 | no | -33.154 | 18.673 | 199 |
| LICHTENBURG | North-West | 410 | 7,006.87 | 1,885.77 | yes | -26.133 | 26.183 | 1491 |
| LOSKOP | Kwazulu-Natal | 272 | 6,570.37 | 1,426.28 | yes | -28.950 | 29.583 | 1167 |
| MAGALIESBURG | Gauteng | 144 | 4,580.97 | 1,539.11 | yes | -26.000 | 27.550 | 1480 |
| MAKOPPA | Gauteng | 36 | 7,128.01 | 955.15 | yes | -26.000 | 27.550 | 1480 |
| MALMESBURY | Western Cape | 104 | 4,484.48 | 1,150.65 | no | -33.276 | 18.706 | 191 |
| MARBLEHALL | Limpopo | 188 | 4,748.69 | 1,043.36 | yes | -24.983 | 29.283 | 915 |
| MATJIESKLOOF | Western Cape | 32 | 5,207.19 | 963.18 | no | -32.683 | 18.883 | 150 |
| MEADOWS | Kwazulu-Natal | 132 | 1,424.24 | 337.57 | no | -30.267 | 29.233 | 1460 |
| MODDERFONTEIN | Western Cape | 56 | 8,005.50 | 899.05 | yes | -32.683 | 18.883 | 150 |
| MODDERIVIER | Western Cape | 232 | 5,535.56 | 1,250.76 | yes | -32.683 | 18.883 | 150 |
| MOORREESBURG | Western Cape | 188 | 4,225.64 | 958.51 | no | -33.154 | 18.673 | 199 |
| NABOOMSPRUIT | Limpopo | 339 | 6,181.27 | 1,274.95 | yes | -24.427 | 28.594 | 820 |
| NAPIER | Western Cape | 100 | 2,982.79 | 907.04 | no | -34.269 | 20.081 | 283 |
| NEWCASTLE | Kwazulu-Natal | 80 | 4,105.20 | 867.41 | yes | -28.137 | 30.315 | 1219 |
| NYLSTROOM | Kwazulu-Natal | 48 | 3,510.21 | 1,024.69 | yes | -24.700 | 28.417 | 1173 |
| OHRIGSTAD | Limpopo | 197 | 6,426.16 | 1,462.26 | yes | -24.719 | 30.562 | 1079 |
| ORANJEVILLE | Mpumalanga | 52 | 6,712.31 | 994.64 | yes | -25.033 | 30.883 | 1005 |
| PETRUSBURG | Free State | 911 | 1,599.36 | 616.64 | no | -29.124 | 25.512 | 1282 |
| PETRUSSTEYN | Free State | 240 | 2,218.75 | 899.76 | no | -28.163 | 28.295 | 1631 |
| PHILADELPHIA | Western Cape | 147 | 4,678.66 | 1,090.02 | no | -33.665 | 18.582 | 71 |
| PIKETBERG | Western Cape | 164 | 3,512.60 | 1,074.08 | no | -32.900 | 18.750 | 274 |
| PLOOYSBURG | Mpumalanga | 24 | 7,348.75 | 860.53 | yes | -26.467 | 29.933 | 1675 |
| POOLS | Western Cape | 196 | 3,396.90 | 1,151.34 | no | -32.797 | 18.888 | 161 |
| PORTERVILLE | Western Cape | 144 | 3,958.54 | 833.28 | no | -33.012 | 18.999 | 149 |
| POTCHEFSTROOM | North-West | 192 | 6,322.94 | 1,171.73 | yes | -26.790 | 26.996 | 1377 |
| PRIESKA | Northern Cape | 636 | 7,332.93 | 1,887.32 | yes | -29.525 | 22.973 | 944 |
| PROTEM | Western Cape | 148 | 3,211.39 | 1,019.88 | no | -34.269 | 20.081 | 283 |
| RAMA | Northern Cape | 791 | 7,784.91 | 1,648.09 | yes | -29.525 | 22.973 | 944 |
| RATELFONTEIN | Northern Cape | 4 | 1,127.50 | 353.40 | no | -31.417 | 20.183 | 1047 |
| REITZ | Free State | 870 | 2,939.00 | 1,351.32 | both | -27.800 | 28.433 | 1615 |
| REMHOOGTE | Western Cape | 626 | 7,801.36 | 2,293.65 | yes | -34.269 | 20.081 | 283 |
| RIEBEEKWES | Western Cape | 32 | 3,406.56 | 1,214.40 | no | -33.154 | 18.673 | 199 |
| RIETPOEL | Western Cape | 36 | 3,196.94 | 1,442.76 | no | -34.252 | 19.735 | 323 |
| RIETRIVER | Northern Cape | 764 | 7,340.28 | 2,277.32 | yes | -29.104 | 24.584 | 1131 |
| RIVERSDAL | Western Cape | 144 | 3,929.75 | 1,091.19 | no | -34.083 | 21.250 | 114 |
| RIVIERSONDEREND | Western Cape | 12 | 4,162.72 | 454.21 | no | -34.162 | 19.907 | 183 |
| RONNEPLEEGTE | Free State | 108 | 1,685.54 | 313.82 | no | -28.950 | 26.350 | 1304 |
| ROODEBLOEM | Eastern Cape | 192 | 4,583.07 | 1,092.39 | no | -32.183 | 24.567 | 808 |
| SAMESUING | Free State | 176 | 2,236.35 | 539.25 | no | -28.950 | 26.350 | 1304 |
| SANDVELD | North-West | 21 | 3,501.90 | 1,137.26 | no | -27.633 | 25.600 | 1231 |
| SANDVET | Northern Cape | 24 | 6,685.83 | 798.82 | yes | -29.104 | 24.584 | 1131 |
| SENEKAL | Free State | 332 | 2,091.72 | 884.60 | no | -28.389 | 27.587 | 1587 |
| SERJANTSRIVIER | Eastern Cape | 72 | 4,271.11 | 1,581.32 | no | -32.183 | 24.567 | 808 |
| SKIETPAD | Eastern Cape | 8 | 4,403.75 | 863.73 | no | -32.183 | 24.567 | 808 |
| SKUINDRIFT | North-West | 161 | 6,837.50 | 2,508.25 | yes | -25.359 | 26.398 | 1000 |
| STANDERTON | Free State | 116 | 5,933.19 | 1,666.60 | yes | -28.163 | 28.295 | 1631 |
| SWELLENDAM | Western Cape | 188 | 3,907.23 | 987.60 | no | -34.033 | 20.450 | 125 |
| TAUNG | Eastern Cape | 72 | 3,538.06 | 1,332.53 | yes | -32.467 | 25.650 | 1198 |
| THEUNISSEN | Eastern Cape | 239 | 6,148.56 | 1,109.70 | yes | -32.467 | 25.650 | 1198 |
| TWEESPRUIT | Free State | 737 | 2,175.98 | 751.44 | no | -29.212 | 27.108 | 1583 |
| TYGERHOEK | Western Cape | 355 | 3,794.51 | 1,131.73 | no | -34.162 | 19.907 | 183 |
| UITKOMS | Eastern Cape | 4 | 2,007.50 | 154.14 | no | -30.767 | 25.583 | 1280 |
| UITVLUG | Western Cape | 139 | 4,530.65 | 1,043.86 | no | -34.083 | 21.267 | 122 |
| UPINGTON | Northern Cape | 624 | 6,998.44 | 1,673.40 | yes | -28.464 | 21.205 | 798 |
| VAALHARTS | Northern Cape | 1,410 | 7,723.55 | 1,977.85 | yes | -27.958 | 24.840 | 1180 |
| VAALWATER | Limpopo | 180 | 5,014.63 | 1,765.21 | yes | -24.300 | 28.117 | 1215 |
| VELDDRIFT | Western Cape | 32 | 4,088.08 | 483.24 | no | -34.083 | 21.267 | 122 |
| VERENA | Limpopo | 24 | 6,645.00 | 1,017.63 | yes | -24.300 | 28.117 | 1215 |
| VILLIERS | Western Cape | 209 | 6,925.45 | 1,559.37 | yes | -33.750 | 19.283 | 670 |
| VOORSTEKOP | Western Cape | 160 | 3,255.67 | 987.55 | no | -34.117 | 20.733 | 250 |
| VREDENBURG | Western Cape | 77 | 2,747.43 | 716.34 | no | -33.067 | 18.350 | 30 |
| VRYHEID | Limpopo | 48 | 3,576.88 | 899.66 | yes | -28.137 | 30.315 | 1219 |
| WESSELSBRON | Free State | 963 | 3,379.83 | 1,312.36 | no | -27.850 | 26.367 | 1325 |
| WINBURG | Eastern Cape | 24 | 1,348.21 | 166.67 | no | -32.467 | 25.650 | 1198 |
| WINTERTON | Kwazulu-Natal | 283 | 4,836.18 | 1,589.01 | yes | -28.883 | 29.489 | 1100 |
| WITSAND | Western Cape | 51 | 3,481.86 | 904.55 | no | -34.083 | 21.267 | 122 |
